# The audience who knew too much: investigating the role of spontaneous theory of mind on the processing of dramatic irony scenes in film

**DOI:** 10.3389/fpsyg.2023.1183660

**Published:** 2023-07-04

**Authors:** Cynthia Cabañas, Atsushi Senju, Tim J. Smith

**Affiliations:** ^1^Cognition in Naturalistic Environments (CINE) Lab, Department of Psychological Sciences, Birkbeck, University of London, London, United Kingdom; ^2^Research Centre for Child Mental Development, Hamamatsu University School of Medicine, Hamamatsu, Japan

**Keywords:** spontaneous theory of mind, dramatic irony, false belief attribution, event comprehension, neurocinematics, film comprehension

## Abstract

As in real life, cinema viewers rely on spontaneous theory of mind (SToM) to interpret characters' mental states. Thus, analyzing cinematic structures offers a unique opportunity to examine ecologically valid sociocognitive processes. We conducted a proof-of-concept study (*N* = 42) to explore how SToM inferences impact film event comprehension in dramatic irony scenes, where knowledge divergence exists between the audience and characters. We hypothesized that spectators would focus more on characters' mental states in such false-belief inducing scenarios compared to scenarios without such disparity. We used six Harold Lloyd silent comedy clips in a narrative comprehension and spontaneous mental state attribution study with a between-subject (Knowledge Manipulation: Installation vs. Control) and within-subject (Phase: Context vs. Exploitation) comparisons. We provided critical information unknown to the characters only to the Installation group and withheld it from the Control group. By comparing differences in participants' descriptions of the clips during the Context phase (varying across groups) and Exploitation phase (same across groups), we evaluated viewers' processing of the same scenes based on their false- or true-belief representations. Our findings indicate that the Installation group used more cognitive mental state words during the Exploitation phase relative to the Context phase, suggesting that exposure to undisclosed critical information enhances the frequency of spontaneous epistemic state inferences and integration into event models of the exploitation. This research advances neurocinematics by highlighting spontaneous sociocognitive processes in event perception and comprehension and provides a novel dramatic irony film corpus and measures for future moment-to-moment SToM processing studies across cognitive-behavioral, physiological, and neural levels.

## 1. Introduction

Have you ever watched a suspense film and found yourself yelling at the screen, warning the protagonist of impending danger? In a heart-pumping scene from Hitchcock's (1960) “Psycho”, as the unsuspecting protagonist, Marion Crane, steps into the shower, the audience is acutely aware of the danger lurking just outside the bathroom door and approaching her. Hitchcock, a master of suspense, frequently used *dramatic irony*—a narrative device where the audience knows something that the characters do not—to heighten the tension and draw the audience deeper into the story. This moment, like many others in film, relies on the viewer's ability to understand the mental states of the characters on screen.

In everyday social situations, we constantly monitor what others know and do not know. For instance, parents often infer their child's knowledge gaps and beliefs to adapt guidance accordingly. This continuous adjustment of our understanding of others' mental states is essential for navigating complex social interactions. As social creatures, we rely on our theory of mind (ToM) to attribute mental states to ourselves and others, allowing us to make sense of differing thoughts and feelings in daily life (Premack and Woodruff, 1978). Importantly, cinema, literature, or theater also makes use of these ToM skills, also known as mentalizing abilities, implicitly motivating us to make sense of characters' actions by attributing and tracking their mental states to understand the stream of events from the narrative (Zunshine, 2006; Levin et al., 2013; Tan, 2013).

Since the emergence of ToM research, a wide range of stimuli has been utilized to study this sociocognitive process, including cartoons, animations, and photographs (e.g., Wimmer and Perner, 1983; Baron-Cohen et al., 1985; Zaitchik, 1990; Abell et al., 2000; Castelli et al., 2000; Gallagher et al., 2000). While these studies have been informative, they have been criticized for both their lack of ecological validity and for the excessive signposting and instruction to produce explicit mental state inferences (Bloom and German, 2000; Dziobek, 2012; Achim et al., 2013). For instance, the Sally–Anne task (Baron-Cohen et al., 1985) is a classic experiment in the field of developmental psychology that tests an individual's ability to understand false beliefs. The task involves presenting the participant with a story in which two characters, Sally and Anne, are present. Sally puts her toy in a basket and then exits the room. While Sally is absent, Anne moves the toy to a box. The participant is then asked to predict where Sally will look for the toy when she returns. The correct answer to pass this false-belief test is that Sally will look for the toy where she last left it, not where Anne moved it.

Despite the Sally–Anne task's foundational role in ToM research, the task has several limitations. One notable concern is its ecological validity, as the task presents a simplified scenario that does not adequately capture the complexity of real-life situations where we often need to integrate contextual information and spontaneously infer others' epistemic states in a more nuanced manner (Wellman et al., 2001; Ruffman and Perner, 2005). Additionally, the Sally–Anne task is primarily designed to assess ToM in young children (Gopnik and Astington, 1988; Astington and Gopnik, 1991), which limits its applicability in studying more advanced ToM abilities in older children and adults. The task may not be sufficiently challenging for older participants including individuals with autism spectrum conditions (ASCs), potentially resulting in ceiling effects or underestimating their ToM capabilities (Apperly, 2011; Senju, 2012; Livingston et al., 2019).

While the Sally–Anne task has its shortcomings, it has served as a critical starting point for research into ToM, particularly in highlighting the importance of false-belief understanding. Due to the simplicity and clarity of false-belief tasks that have allowed for more controlled experimentation, ToM research in adults has tried to adapt false-belief tasks for adults to understand the underlying cognitive mechanisms of this complex sociocognitive process. For instance, researchers have attempted to examine the curse of knowledge bias (Birch and Bloom, 2007; Bernstein et al., 2011; Sommerville et al., 2013) and higher-order ToM understanding (Kinderman et al., 1998; Stiller and Dunbar, 2007; Oesch and Dunbar, 2017). However, it remains unclear whether these tasks are adequate for exploring the intricacies of adult ToM as they may require more advanced conceptual knowledge or be influenced by working memory and executive function capacity (Brown-Schmidt, 2009; Ryskin and Brown-Schmidt, 2014). Addressing these concerns and identifying more suitable tasks are crucial for advancing adult ToM research and understanding individual differences in everyday ToM abilities.

The development of tasks that incorporate such false-belief structures together within naturalistic stimuli, such as films, could be a step in this direction as they require the integration of contextual information and the understanding of multiple mental states simultaneously (Levin et al., 2013; Tan, 2013). This approach may provide a more ecologically valid assessment of mentalizing abilities while still maintaining experimental control. In this study, we propose a novel approach to studying ToM by harnessing the engaging power of films featuring dramatic irony structures, which could potentially serve as a naturalistic, filmed adaptations of false-belief tasks.

The use of dramatic irony often follows a three-act structure (Lavandier, 2005 modified and extended to include Cohn, 2016): (1) An *establisher* scene sets up the situation and introduces the characters' goals. (2) An *installation* scene provides crucial information that one or more characters are unaware of, which sets the stage for the dramatic conflict to come. These oblivious characters are known as the victims of dramatic irony. Finally, (3) the *exploitation* scenes depict the victims' reactions and actions in response to their ignorance, which can lead to misunderstandings, decoys, or deception.

The structure of this scenario closely resembles that of the classical Sally–Anne task, but it is integrated with additional contextual information. For instance, in the mentioned iconic shower scene from “Psycho” first exhibits Marion's vulnerability as she is preparing to take a shower (*establisher*). While Marion, is under the water in the shower, the audience is able to catch a glimpse of shadow behind the curtain in the shower (*installation*). The audience quickly understands that Marion, the victim of dramatic irony, is unaware of the presence of this figure. As the shadow slowly approaches, it starts taking form into what seems an old lady prepared to violently attack Marion with a knife (*exploitation*), while the audience helplessly anticipates the consequences of Marion's false belief that she is alone in the bathroom.

While the field that studies theory of mind has historically centered around the investigation of (false) belief attributions, scholars such as Phillips et al. (2021) have recently highlighted the importance of examining the role of knowledge attributions in social interactions. Critically, dramatic irony creates a unique opportunity to distinguish between when viewers categorize a character as being ignorant and when they label them as holding false beliefs (Scott and Baillargeon, 2009; Baillargeon et al., 2010). In particular, when labeling characters as ignorant, we have to attribute lack of knowledge to them, whereas when attributing false beliefs, we assume they hold (incorrect) information not supported by reality. The temporal and contextual factors that influence individuals' tendency to make knowledge attributions are currently unknown.

On the contrary, over the past decade, many social neuroscience researchers have shifted toward using films as a rich source of naturalistic stimuli, enabling the examination of ToM processing in more realistic scenarios (Dziobek, 2012; Achim et al., 2013; Devine and Hughes, 2013). However, even studies that use audio-visual stimuli often examine explicit theory of mind, where participants are manifestly asked to infer mental states of characters in the film (Heavey et al., 2000; Dziobek et al., 2006; Golan et al., 2006; Devine and Hughes, 2013). In these tasks, it is assumed that individuals spontaneously engage in theory of mind reasoning during such scenarios and in everyday life [see Heyes (2014) for an argument about submentalizing]. However, there is a challenge to test such assumption, given that current tasks typically instruct participants to mentalize.

The need to overcome this critical limitation led to the development of implicit or spontaneous theory of mind (SToM) tasks that aimed at measuring the ability to infer mental states in naturalistic scenarios without explicit prompts or instructions. Some of these strategies include free-viewing paradigms combined with talk aloud tasks or *post-hoc* free recall comprehension questions which researchers can code the mentalizing skills and tendencies of participants (Klin, 2000; Barnes et al., 2009; Rice and Redcay, 2015; Altschuler et al., 2018; Bálint et al., 2018; Rooney and Bálint, 2018). Importantly, Apperly (2012) distinguishes between the ability to mentalize and the tendency to spontaneously pay attention to another person's mental states. This distinction is essential in SToM paradigms since there is increasing evidence that individuals with ASC may perform successfully in mentalizing tasks attending to socially relevant information when explicitly instructed to but might be less likely to mentalize spontaneously without explicit instruction or task demand (Senju, 2012; Dufour et al., 2013).

Several studies indicate that examining how and what we understand from a film narrative has the potential to reveal differences in mentalizing tendencies. For instance, Lahnakoski et al. (2014) observed differences in eye movements when viewers shifted their focus between characters and objects, while Yeshurun et al. (2017) found that neural representations of movie clips were more similar within groups who shared the same beliefs about a situation.

In this study, we present a proof-of-concept demonstration of the value of utilizing dramatic irony sequences in film as a naturalistic test of viewers' complex SToM processing. Our main goal was to examine whether dramatic irony structures naturally prompt audiences to make more inferences about characters' epistemic states and beliefs compared to control scenes without dramatic irony. We propose that by investigating the processing of these structures in films through a free recall task, we can gain valuable insights into individuals' spontaneous mental state inferences. This approach offers a point of reference, illustrating typical responses to the task and stimulus, and lays the methodological foundation for future investigations into the neural basis and individual differences in these processes.

In dramatic irony scenes, relevant information about the characters' ignorance and/or false beliefs is presumably extracted from the *installation* scene and integrated into a situational event model or “person schema” to understand characters in films (Smith, 1995; Zwaan and Radvansky, 1998; Loschky et al., 2020), drawing on their knowledge of real people. Moreover, Bálint et al. (2018) argued that by increasing the attentional resources allocated to characters and their facial expressions, close-ups could potentially boost the likelihood that a viewer's mental model of a narrative includes the mental states of the characters. Both cognitive ToM (recognizing others' beliefs, thoughts, and motivations) and affective ToM (inferring their emotions and feelings) are essential for understanding the divergence between our own beliefs and emotions and those of the victim of dramatic irony. For instance, in the “Psycho” example only by considering and incorporating Marion's beliefs into an event model of the scene, we can make sense of her calm, untroubled emotional expression in the shower while we see a threatening figure behind her.

Thus, we hypothesized that, similarly, by increasing attentional resources to the salient disparity of knowledge between character and audience, the structure of dramatic irony scenes (vs. control scenes) would prompt spectators to infer more often the mental state of characters, both cognitive and affective, and incorporate them into their event models. We examined this hypothesis by manipulating the audience's access to knowledge from the *installation* scenes in a narrative comprehension and spontaneous mental state attribution study, allowing us to compare how viewers process the same scenes depending on their ToM representations.

Participants in the Installation group watch the installation scene which contains crucial information to understand the dramatic irony conflict, while those in the Control group do not. We measured comprehension of the dramatic irony conflict and the frequency of mental state references to examine how each condition determined how participants reasoned about the events and described them. Critically, including both complementary measures can provide a comprehensive and nuanced understanding of how theory of mind is involved in the comprehension of dramatic irony.

Previous studies have used coding schemes that often identify both affective mental states, which refers to others' emotions or feelings (e.g., “*Marion looks relaxed and undisturbed in the shower*”), and cognitive mental states, which refers to others' thoughts, beliefs, or intentions (e.g., “*Marion thinks she is alone in the bathroom*”) (Klin, 2000; Rice and Redcay, 2015; Altschuler et al., 2018; Rooney and Bálint, 2018). However, these types of mental states were previously collapsed together for later analysis. Crucially, there is a large body of evidence that shows that emotional and cognitive components of sociocognitive processes such as ToM and empathy are interdependent but separate mechanisms in the brain (Dziobek et al., 2008; Abu-Akel and Shamay-Tsoory, 2011; Zaki and Ochsner, 2012). Although ToM and empathy are distinct sociocognitive processes, some overlap exists in their definitions among various authors, particularly when comparing cognitive aspects of ToM and empathy with affective aspects of ToM and empathy. Nevertheless, empathy involves an experience-sharing component that is not necessarily inherent in ToM. Preckel et al. (2018) highlight that cognitive and affective empathy, as well as theory of mind (ToM), are underpinned by distinct, independent brain networks, while also acknowledging the interplay between these processes. Specifically, Cuff et al. (2016) note that while some empathy definitions focus on either affective or cognitive aspects, many encompass both. The authors further support this distinction by citing empirical evidence from research in personality, developmental disorders, and neurological studies, supporting the notion that cognitive and affective empathy are separate constructs. Specifically during film watching, Shany et al. (2021) found different neural patterns for affect sharing, affective ToM, and cognitive ToM. To capture these dissociative components in the processing of dramatic irony scenes, we considered cognitive and affective mental states both separately and together in our analysis.

We predicted that participants in the Installation group would understand the dramatic irony conflict that arises from the victim's ignorance of critical information more often than participants in the Control group. This prediction served as a manipulation check that exposure to *installation* scene is required to understand dramatic irony and that the *exploitation* scene alone does not contain sufficient information.

We expected the Installation group to use a higher frequency of overall mental state references (H1) than the Control group, in line with previous literature which demonstrated that increased number of mental state references in free recall was associated with more accurate mental state attribution (e.g., Bálint et al., 2018; Rooney and Bálint, 2018). We predicted participants in the Installation group would show a higher frequency of cognitive mental state references (e.g., beliefs, thoughts, and intentions) in their free recall responses compared to the Control group (H1.1), suggesting that exposure to critical information unknown to a character, promotes a more thorough understanding and integration of that characters' thought processes and mental perspectives. We also anticipated participants in the Installation group would demonstrate a higher frequency of affective mental state references (e.g., emotions, feelings, desires) vs. the Control group (H1.2), indicating that experiencing the installation scene enhances one's sensitivity to the characters' emotional experiences and the subtleties of their affective states.

Finally, to account for the difference in clip length between the Installation and Control groups, we examined the frequency of mental states specifically in participants in two different phases of the descriptions: the Context phase and the Exploitation phase (see Section 2.5). We did not expect to find differences in mental state references in the Context phase but expected the Installation group to differ from the Control group in the number of overall (H2), cognitive (H2.1), and affective (H2.2) mental state references in participants' descriptions of the Exploitation phase, where the dramatic irony conflict occurs.

## 2. Materials and methods

### 2.1. Design

The present study was an online experiment conducted on Gorilla.sc (Anwyl-Irvine et al., 2020) with a mixed-design: a between-subject variable (*Knowledge Manipulation*) with two levels (Installation vs. Control); a within-subject variable (*Phase*, which here denotes the part of the description that participants referred to) with two levels (Context vs. Exploitation) and two dependent variables, dramatic irony conflict comprehension (DIcomp) score and mental state reference frequency (MSRF) as a proxy of SToM tendency. Participants were randomly assigned to one of the two Knowledge manipulation conditions. The order of the presentation of the six clips (blocks) was randomized.

### 2.2. Participants

A convenience sample of 50 participants (33 female participants, age: M = 30, SD = 9.24) was recruited from the university student participant pool (SONA). Given that there were no previous studies we could use to power this study, the target sample size (*N* = 42) was derived from an a priori power analysis carried out using the software G^*^Power (Faul et al., 2009) for an estimated effect size of Cohen's d = 0.8 with sufficient power (0.9; α = 0.05). Given the novelty in the experimental online design, the dropout rate for this study was uncertain. The initial sample size of 50 participants was chosen to compensate for the anticipated dropout rate due to exclusion criteria or technical errors.

The mean age of our sample (30 years old) and the large standard deviation is reflective of the diverse student population at our university, which includes a high proportion of mature and international students. Therefore, inclusion criteria consisted of normal or corrected-to-normal vision, an advanced English level to answer the comprehension questions and no previous diagnosis of autism spectrum conditions (ASCs). Given the limited number of trials (six clips), participants were excluded if they did not answer the free recall comprehension tasks for each of the six clips. They were also excluded if their descriptions for each clip did not include at least 1 sentence per fragment (2 for the Control group since these participants see *establisher* and *exploitation* scenes; 3 for the Installation group since these participants see *establisher, installation*, and *exploitation* scenes) demonstrating low effort in performing the task. To avoid un-blinding of conditions, this exclusion phase was performed by author CC, before the coding of comprehension and mental state references was performed by two independent coders (BS and EE) unaware of the nature of the task and the groups these participants belonged to.

From the final sample, eight participants were discarded: five for not completing all the measures or due to previously set exclusion criteria for data quality, one for having previously seen one or more of the films, and two for reporting an intermediate or lower English level. Experimental procedures were approved by Birkbeck, University of London Ethics Board (181949). All subjects provided written informed consent.

### 2.3. Film clip corpus design

Stimuli were six self-contained film excerpts taken from different Harold Lloyd comedy silent movies (U-certified). The videos convey a short storyline, with no sound, white text is presented on a black background in between scenes (intertitles with verbal information). The content of each clip with an illustrative still of each phase can be found in the Supplementary material. All of the films the clips were taken from were silent-era Harold Lloyd films, including “Never Weaken” (Newmeyer, 1921), “Girl Shy” (Newmeyer and Taylor, 1924), “The Freshman” (Newmeyer and Taylor, 1925), “For Heaven's Sake” (Taylor, 1926), and “The Kid Brother” (Wilde et al., 1927). This selection was inspired on the Silent Film task developed by Devine and Hughes (2013), who used silent comedy clips from a Harold Lloyd single film. The Silent Films task is designed to measure participants' explicit understanding of beliefs and desires and engaging for a broader audience including adults and older children with different language groups and children who may have low verbal ability. We aimed to build on these stimulus design criteria by selecting similar Harold Lloyd film clips albeit in a systematic way based on the identification of false-belief inducing situations in dramatic irony structures, with the intention to create a film corpus which could be used for future studies examining implicit measures of SToM.

There are several other benefits to this selection: First, given that these films are approximately a century old, it is very unlikely that participants have watched them; second, the silent film format controlled for the influence of audio or verbal information, focusing viewer's on the images as the main source of information and making the stimuli suitable across future differences in participant verbal IQ (e.g., in studying Autism); and third, given the canonical structure of dramatic irony described in the introduction (Lavandier, 2005), the identification of these scenes (*establisher, installation, and exploitation*) could allow us to make testable hypothesis about temporal dynamics and sub-processes of SToM.

**Figure 1:**
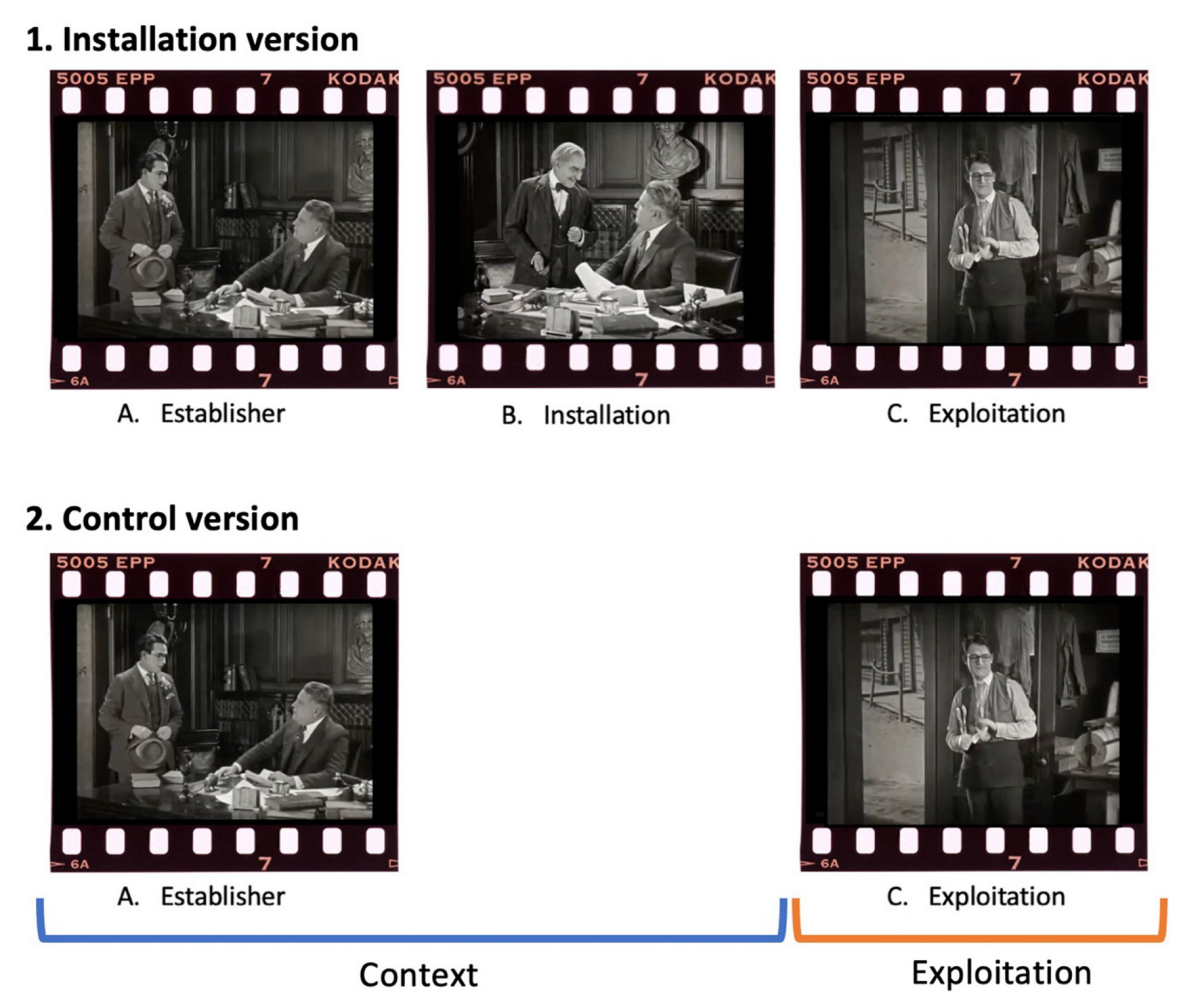
Example stills of both conditions from “Girl Shy” (Newmeyer and Taylor, 1924) included in the film corpus. In the Installation version, **(A)** Establisher: Harold visits a publishing house to inquire about the possibility of publishing his book. However, the publisher finds his book to be extremely comical, so they reject it and inform Harold that he will receive a rejection letter in the mail. **(B)** Installation: When Harold leaves, a senior employee convinces the editor to reconsider and publish the manuscript as a comedy. He then instructs the employee to send a check to Harold instead of the rejection letter. **(C)** Exploitation: Harold, downhearted and unaware of the content of the letter, tears it apart without opening it. 2. The control version only has **(A)** Establisher and **(C)** Exploitation; therefore, participants should interpret that Harold tore apart the rejection letter. The approximate duration of the clips was around 3 min. Stills taken with permission from Girl Shy (1924) © 2023 Harold Lloyd Entertainment, Inc. Reproduced with permission.

Two versions of the clips were created: The Installation complete dramatic irony film clips composed of three scenes (*establisher* scene, *installation* scene, and *exploitation* scene) creating a divergence of beliefs between participants and characters, thus, depicting instances of deception and misunderstanding. To manipulate the knowledge context, in the Control version of the clips, the *installation* scene is edited out; therefore, participants only see two scenes: *establisher* scene and *exploitation* scene. For this purpose, we made sure that the characters' behaviors were congruent or plausible in Installation and Control conditions. A short description and the duration for each version can be found in Table 1. Critically, viewers in both groups watch exactly the same *exploitation* scenes, allowing us to compare how viewers process the same scenes when they know more than the victim vs. when they do not (they are as ignorant as the victim). Figure 1 provides a visual representation of both Installation and Control versions of the film clips for illustration purposes. Detailed copyright information and permissions for the reuse of these clips can be found on our dedicated project webpage: https://www.cinelabresearch.com/haroldlloydproject. Researchers interested in using these stimuli for their studies are encouraged to contact us directly through the form provided on the website for more information.

**Table 1:** Summary of film clips with respective control and installation versions, including their duration and a brief description of the scenes.

**Clip title**	**Version**	**Duration**	**Short description**
Never Weaken I	Control	2 m 21 s	Harold aims to showcase an osteopathic clinic's effectiveness by healing a man on the street, attracting new clients to the clinic where his love interest works
	Installation	3 m 26 s	Harold devises a plan with an acrobat to stake a fake injury recovery, drawing the attention of potential clients to the osteopathic clinic where his love interest works
The Freshman	Control	3 m 26 s	Harold attempts to join the football team, successfully secures a spot, and enthusiastically heads to the field to play
	Installation	3 m 54 s	Harold excitedly tells a girl he made the team and eagerly goes to the field, not knowing that his real role is the water boy
Never Weaken II	Control	1 m 09 s	Harold proposes to a girl and overhears a conversation where he finds out that she is being proposed to by another man
	Installation	1 m 25 s	Harold proposes to a girl who accepts, but later misunderstands her conversation with her brother, thinking she's being proposed to by another man
Girl Shy	Control	2 m 26 s	Harold tries to publish his book but is rejected by the publisher and receives a rejection letter in the mail
	Installation	3 m 06 s	Harold attempts to publish his book, and although initially rejected, the publisher reconsiders. Harold, believing the letter contains a rejection slip, tears up the unopened envelope containing a check
For Heaven's Sake	Control	4 m 11 s	A missionary and his daughter write to Harold for help raising money for their mission. Harold comes across their mission cart and offers them a significant contribution
	Installation	5 m 14 s	Harold accidentally burns a mission cart, writes a check to compensate, but is mistaken for a generous donor for the mission
The Kid Brother	Control	1 m 32 s	Two men trick the sheriff into signing a permit for their traveling show
	Installation	5 m 06 s	Harold, dressed as his sheriff father, is tricked into signing the permit for the two men

### 2.4. Procedure

Participants run the experiment through their web browser through an online experimental task engine (Gorilla.sc) used to ease participants access to the experiment at home. Immediately after watching each clip, we asked them to perform a free recall task by answering to a prompt on the screen saying, “Please, take about five minutes and write a paragraph about what happened in the clip you just saw and why it happened.” Participants typed their responses in a text box. There was no time limit to answer to this prompt. Participants were not asked for specific aspects of characters' mental states to avoid potential bias allowing us to obtain only SToM responses (Barnes et al., 2009; Rice and Redcay, 2015; Bálint et al., 2018) and measure potential differences between conditions. The approximate total duration of the task for each group was ~21 min for the Installation group and 15 min for the Control group. These durations ensured that participants in both Knowledge conditions were exposed to an adequate amount of content while keeping the overall task duration manageable. To prevent fatigue and ensure participant engagement, breaks were provided after each block, allowing participants to rest before continuing with the subsequent clips.

At the end of the experiment, they were asked whether they had seen any of the films and a short debrief question about whether they noticed any pattern across the clips (i.e., the dramatic irony structures) to ensure that they were not aware of the dramatic irony structures, potentially having an influence on their SToM. None of the participants reported having noted a pattern in the structure of the clips. Finally, a debrief was shared with the participants explaining the background of the study and what had been measured as part of the study.

### 2.5. Coding scheme

Free recall responses were coded by an independent blind researcher for the comprehension of dramatic irony conflict and the frequency of mental state use (see next section for measure definitions). To ensure inter-rater reliability, a second researcher coded 25% of a sample of descriptions at random. Initial inter-rater reliability was evaluated for both dramatic irony comprehension (DIcomp) and Overall, Cognitive, and Affective mental state reference frequency (MSRF) coding separately using Krippendorff's alpha, which was calculated to be 0.938 for DIcomp; 0.749 for Overall MSRF; 0.713 for Cognitive MSRF; and 0.725 for Affective MSRF, which are considered acceptable or above levels of agreement (Krippendorff, 2004).

#### 2.5.1. Dramatic irony conflict comprehension

Using a grading scheme based on Barnes and Baron-Cohen (2012) and Lavandier (2005), participants are scored on their understanding of dramatic irony conflict in a narrative. Full understanding (2 points) requires explaining the victim's ignorance of critical information and its impact on their goals. Partial understanding (1 point) involves recognizing the victim's ignorance but not its consequences. Failed understanding (0 points) lacks any reference to the victim's ignorance or its impact. In the “Girl Shy” example (see Table 1), mentioning Harold's unawareness of the check earned partial understanding, while discussing how this relates to his goal to earn money for publishing his book earned full understanding. No mention of his ignorance or its impact resulted in failed understanding. Since participants watched six clips, the possible total scores for dramatic irony conflict comprehension ranged from 0 to 12.

#### 2.5.2. Mental state reference frequency

Based on the ToM coding scheme by Bálint et al. (2018), informed by Meins and Fernyhough (2006), participant descriptions were divided into subject–verb–predicate coding units. Coders identified explicit mental state references. Here, mental state reference was defined as “any reference to an individual's mental life, relating to desire, wish, emotion, will, mind, imagination, interest, intellect, or metacognition” (Bálint et al., 2018). These references were also categorized as (a) affective (e.g., feelings and desires) or (b) cognitive (e.g., memory, knowledge, and intention). To account for individual differences, participants received scores for the proportion of mental state references to total coding units, indicating their theory-of-mind responding level.

At a second coding stage, to account for the difference in clip length between the Installation and Control groups, a third blind coder identified sentences referring to Exploitation scenes to separate scores for frequency of affective and cognitive mental states in Context and Exploitation phases, relative to coding unit count. The first phase included the description of the Context, which differs per group, including the establisher and installation scenes in the Installation group and only the establisher scene in the Control group, since the latter did not watch an installation scene. We did not expect to find differences in mental state references in this phase. The second phase was composed of the description of the exploitation scenes, which are the scenes that are viewed in both Installation and Control groups (see Figure 1), where we did expect to find differences in MSRF. The primary responsibility of this coder was thus to identify when participants' descriptions started referring to the exploitation scene (Exploitation phase), which was clearly defined in the coding manual. The parts of participant descriptions not belonging to the Exploitation scene were categorized as Context. This task did not involve the interpretation of ambiguous mental states or the assessment of participants' understanding of conflicts but rather focused on a more straightforward identification process based on well-defined criteria.

## 3. Results

This analysis plan for this study was preregistered on the Open Science Framework [10.17605/OSF.IO/PQRU6] Additional analyses examined the relationship between dependent variables (DIcomp and MSRF) for the Installation group. R in R-studio was used for data management and statistical analysis, ensuring assumptions of normality and homogeneity of variances were met for *t*-tests and ANOVAs.

Overall MSRF and Cognitive MSRF were normally distributed, allowing parametric testing. Affective MSRF was slightly non-normal and positively skewed. As a result and to further validate our findings, we carried out both parametric tests using logarithmically transformed data and non-parametric tests, aiming to demonstrate the robustness and consistency of our results regardless of the specific statistical test employed (Field et al., 2012). Levene's test confirmed homogeneity of variances (*p* > 0.05). Table 2 displays summary statistics for measured variables per knowledge condition across all clips.

**Table 2:** Descriptive and inferential statistics of all measures by Knowledge condition.

**Measures**	**Condition**	**Mean**	**SD**	**95% CI lower**	**95% CI upper**	***t*-test**	***p*-value**
					
Age	Control	29.00	8.16	25.18	32.82	−0.564	0.576
	Installation	30.52	9.11	26.38	34.67	-	-
Gender	Control	1.75	0.44	1.54	1.96	0.575	0.568
	Installation	1.67	0.48	1.45	1.89	-	-
English Level	Control	4.30	0.86	3.90	4.70	−0.309	0.758
	Installation	4.38	0.80	4.01	4.75	-	-
DIcomp	Control	1.75	1.59	1.01	2.49	−13.368	**0.000**
	Installation	9.71	2.19	8.72	10.71	-	-
Overall MSFR	Control	37.11	14.38	30.38	43.84	−1.476	0.149
	Installation	42.70	9.13	38.54	46.85	-	-
Cognitive MSFR	Control	21.65	8.84	17.51	25.78	−3.291	**0.002**
	Installation	31.32	9.97	26.78	35.86	-	-
Affective MSFR	Control	15.47	7.86	11.79	19.15	1.922	0.063
	Installation	11.48	5.06	9.17	13.78	-	-

DIcomp, Dramatic Irony Comprehension; MSRF, Mental State Reference Frequency; SD, standard deviation; CI, confidence interval.

Bold values denote significance at the level p ≤ 0.05.

### 3.1. Manipulation check: DIcomp in installation vs. control groups

As a preliminary test, we assessed our manipulation check (higher DIcomp in the Installation group than Control) using a mixed-design ANOVA with Knowledge manipulation (Installation vs. Control) as a between-subject factor and Clip (1–6) as a within-subject factor and including participants' number in the error term. This accounted for individual variation and allowed us to similarity across clips in DIcomp. Significant main effects were found for Knowledge condition (*F* (1.234) = 344.44, *p* < 0.001, partial η^2^ = 0.60) and Clip (*F* (5.1170) = 2.97, *p* = 0.013, partial η^2^ = 0.11), with a significant interaction between them (*F* (5.1170) = 8.96, *p* < 0.001, partial η^2^ = 0.04), indicating that the effect of Knowledge condition differed depending on the clip being shown.

**Figure 2:**
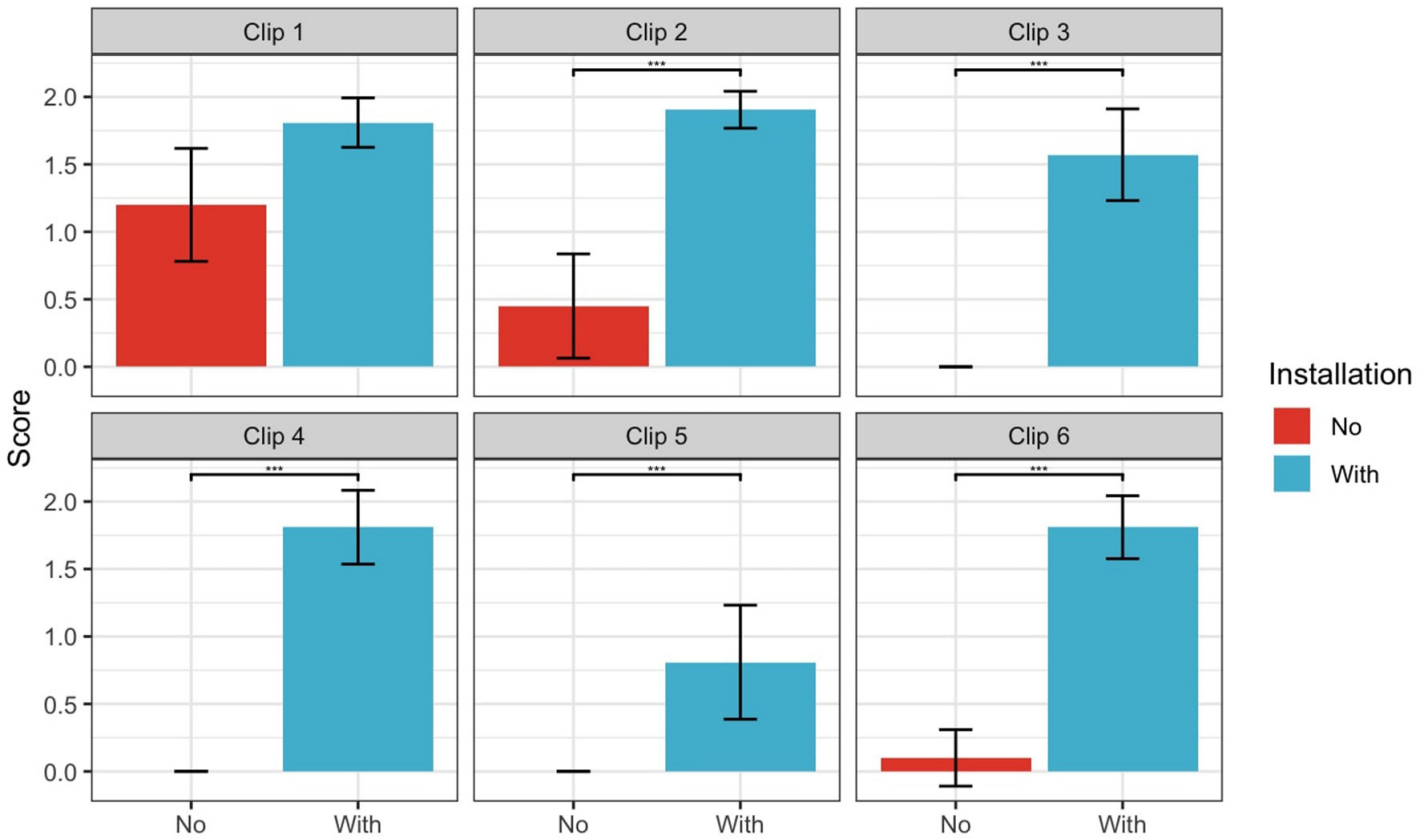
Dramatic Irony Comprehension (DIcomp) scores for each clip separately. Error bars represent 95% confidence intervals.

We conducted six Welch's *t*-tests to compare DIcomp scores between groups for each clip. The results indicated significantly higher scores in the Installation group for five out of six clips (all *p* < 0.05), though the effect varied. As shown in Figure 2, Clip 1 showed no significant difference after Bonferroni correction (*p* = 0.073).

### 3.2. Hypothesis 1: MSRF in installation vs. control groups

**Figure 3:**
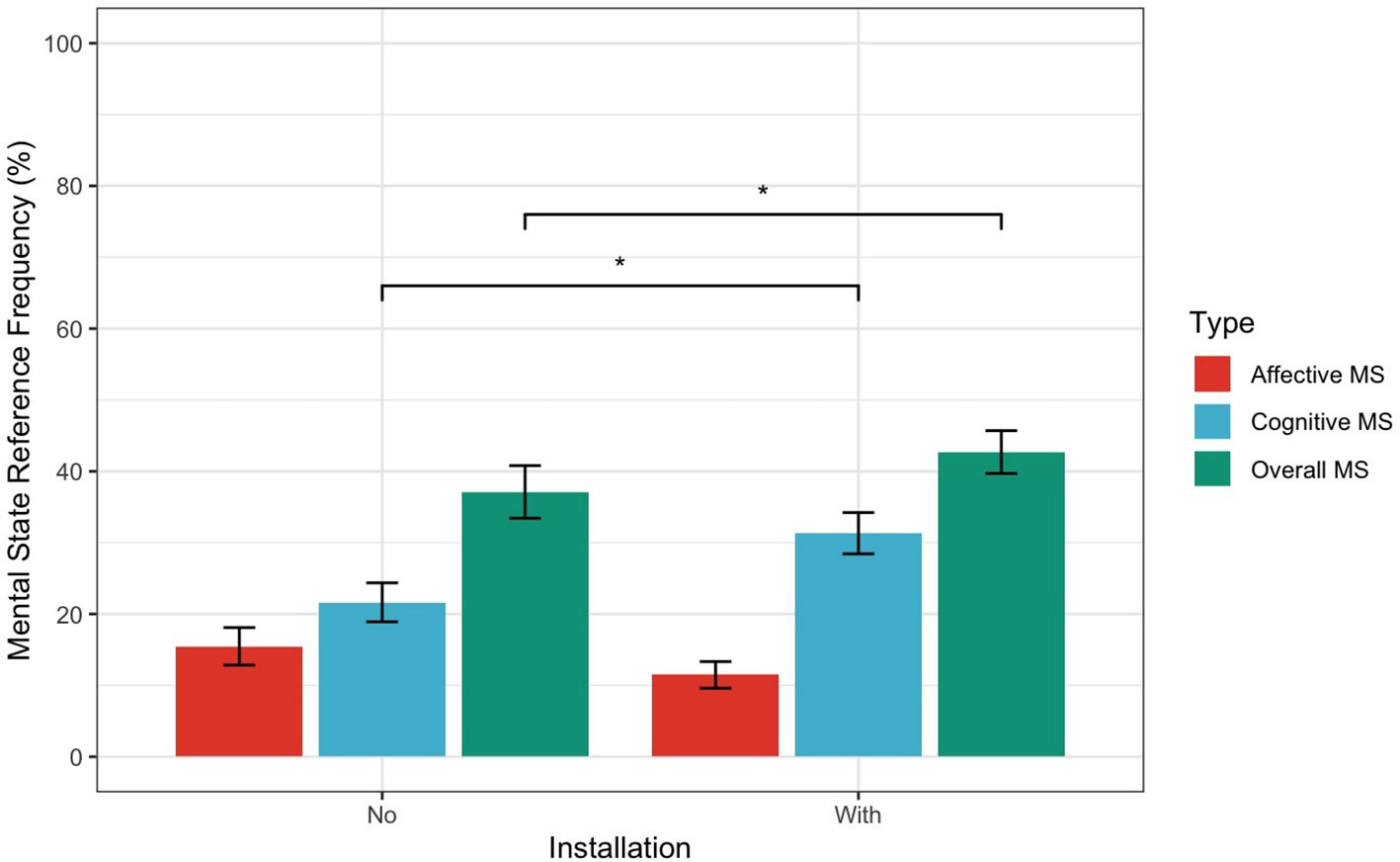
Comparison of Mental State Frequencies by condition across clips. Error bars represent 95% confidence intervals. ^*^Indicates statistical significance with a *p*-value less than 0.05.

To test H1 (higher MSRF in the Installation group than Control), a mixed-design ANOVA assessed the effects on MSRF of Knowledge manipulation (Installation vs. Control) as a between-subject factor, Clip (1–6) as a within-subject factor, and participants' number in the error term. By doing so, we accounted for individual variation and investigated the similarity across clips in MSRF. As shown in Figure 3, the results indicated a significant effect of condition on Overall MSRF (*F* (1, 228) = 5.197, *p* = 0.0236) and Cognitive MSRF (*F* (1.228) = 25.217, *p* < 0.0001). On the contrary, the effect of condition on Affective MSRF was not significant (*F* (1.228) = 3.457, *p* = 0.063). Logarithmically transforming and carrying out non-parametric, the data did not affect the results of the statistical tests so here we only report the parametric tests.

There was a significant effect of Clip on Cognitive MSRF (*F* (5.228) = 2.555, *p* = 0.0284) and Affective MSRF (*F* (5.228) = 5.066, *p* = 0.0002) but not on Overall MSRF (*F* (5.228) = 1.176, *p* = 0.3215). The interaction between condition and Clip was not significant in any of the analyses, suggesting that the effects of Knowledge manipulation on Overall, Cognitive, and Affective MSRF did not vary across different clips. These preliminary tests were conducted to ensure that the MSRF was consistent across the six clips included in the study. Partial eta-squared ($\eta_{\text{p}}^{2}$) effect sizes for Overall, Cognitive, and Affective MSRF were 0.02, 0.10, and 0.04 for Knowledge condition and 0.03, 0.05, and 0.10 for Clip, respectively.

### 3.3. Hypothesis 2: DIcomp and MSRF in context vs. exploitation phase across groups

To test H2 (higher MSRF in Exploitation phase than in the Context only in the Installation group and not in the Control), we conducted a 2x2 mixed ANOVA assessing the effect of knowledge manipulation (Installation vs. Control) on mental state reference frequency (MSRF) in Exploitation vs. Establisher phases. Overall MSRF and Affective MSRF were normally distributed, while Cognitive MSRF was slightly skewed but reasonably symmetrical (see Supplementary Figure) to conduct ANOVA which is robust to non-normality with a large enough sample size. We conducted the same analysis transforming the data by squared rooting the cognitive MSRF values, to check for consistency as recommended by Field et al. (2012). All effects are reported significant at a *p*-value of < 0.05.

**Figure 4:**
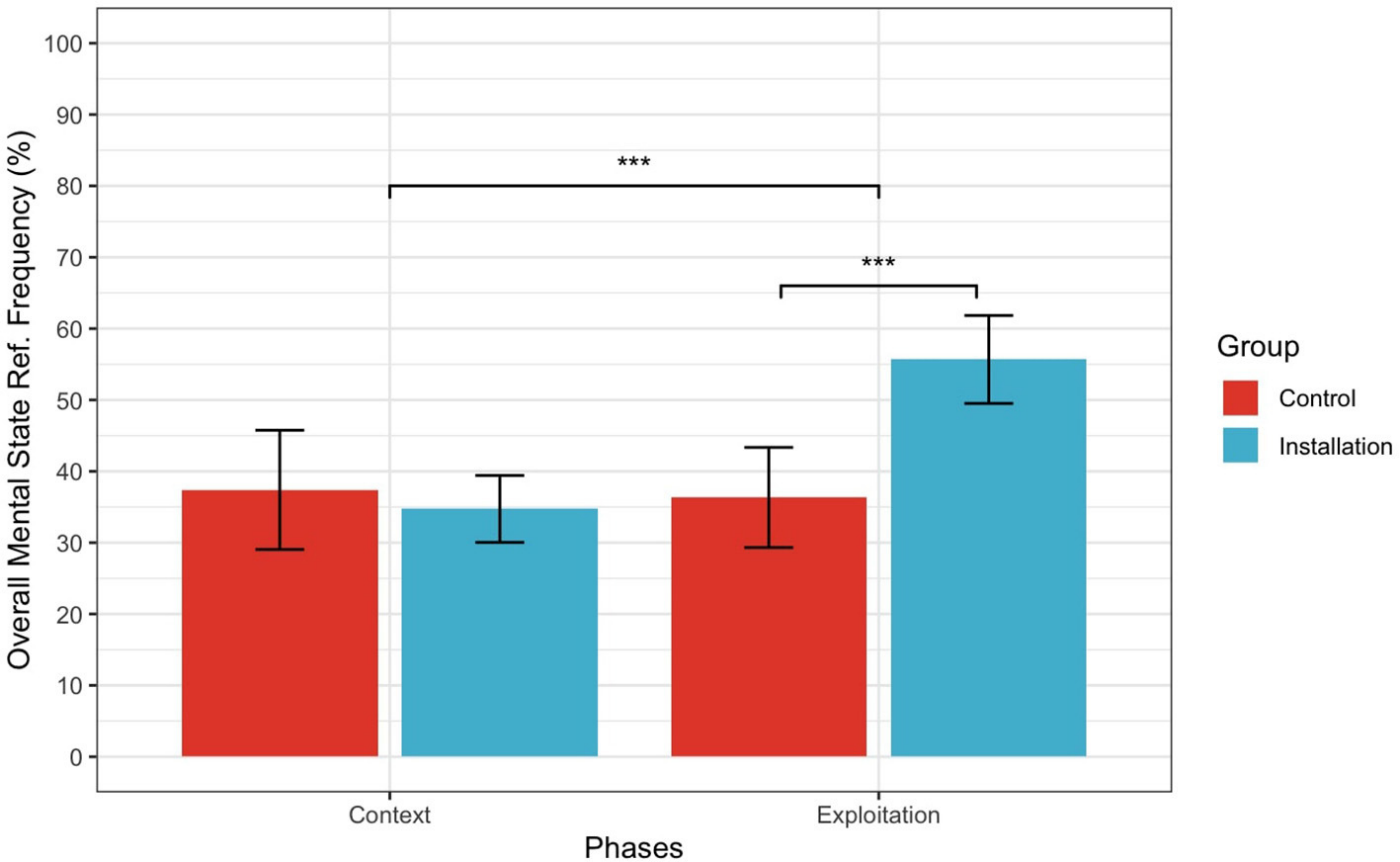
Interaction between Phase and Condition for Overall Mental State Frequency. Error bars represent 95% confidence intervals. ^*^Indicates a *p*-value less than 0.05, ^*^^*^Indicates a *p*-value less than 0.01, ^*^^*^^*^Indicates a *p*-value less than 0.001.

For H2.1 (Overall MSFR), there was a significant main effect of Knowledge condition (*F* (1.39) = 4.67, *p* = 0.036, $\eta_{\text{p}}^{2}$ = 0.10) and Phase (*F* (1.39) = 18.67, *p* < 0.001, $\eta_{\text{p}}^{2}$ = 0.32), with a significant interaction (*F* (1.39) = 22.90, *p* < 0.001, $\eta_{\text{p}}^{2}$ = 0.37). As shown in Figure 4, this significant interaction shows that participants in the Installation group used significantly more Overall MSRF when describing the Exploitation phase compared to Context phase; however, this was not the case for participants in the Control group.

**Figure 5:**
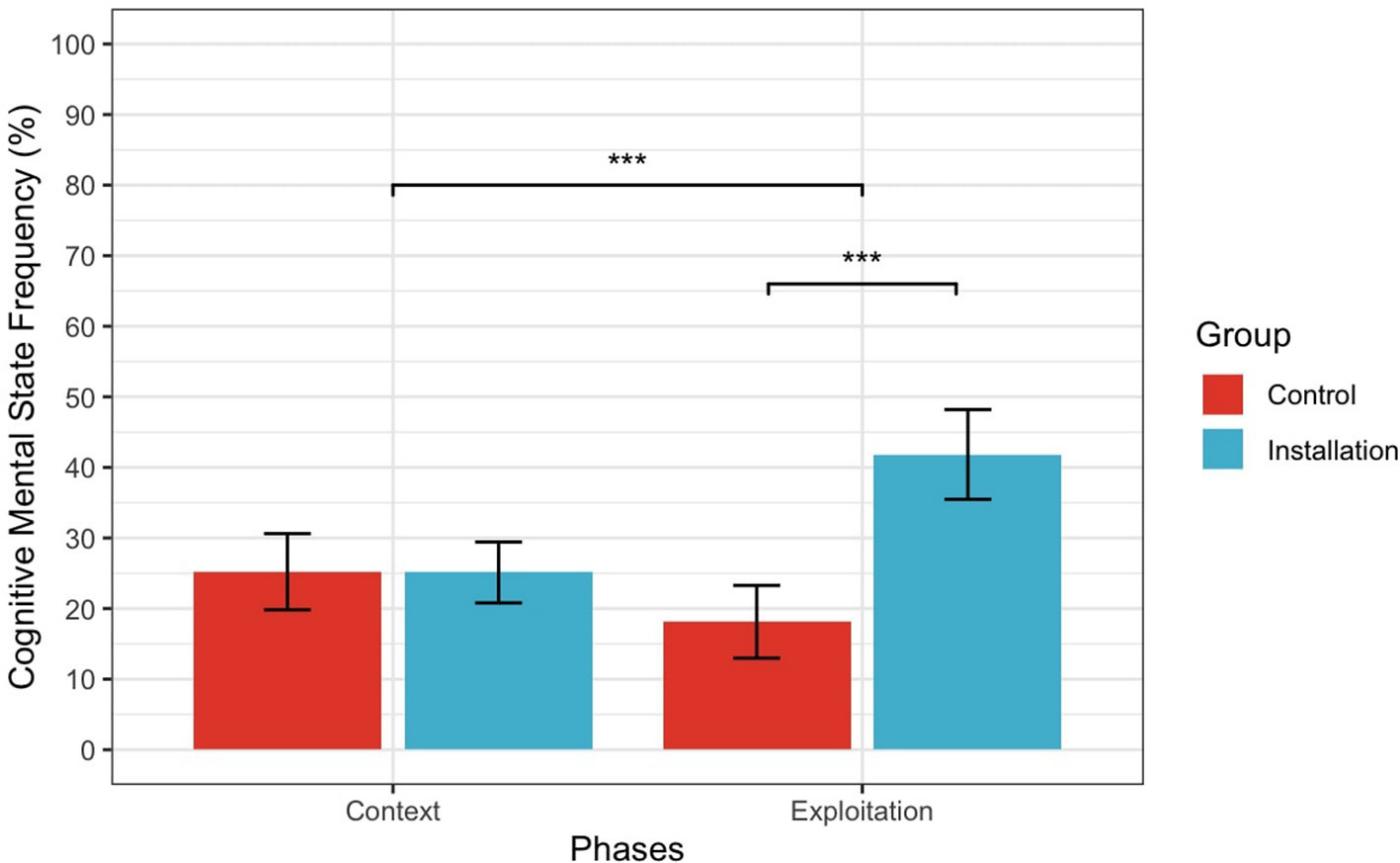
Interaction between Phase and Condition for Cognitive Mental State Frequency. Error bars represent 95% confidence intervals. ^*^Indicates a *p*-value less than 0.05, ^*^^*^Indicates a *p*-value less than 0.01, ^*^^*^^*^Indicates a *p*-value less than 0.001.

For H2.2 (cognitive mental states), there was a significant main effect of Knowledge manipulation (*F* (1.39) = 15.42, *p* < 0.001, $\eta_{\text{p}}^{2}$ = 0.28) and Phase (*F* (1.39) = 5.57, *p* = 0.023, $\eta_{\text{p}}^{2}$ = 0.21), with a significant interaction (*F* (1.39) = 34.12, *p* < 0.001, $\eta_{\text{p}}^{2}$ = 0.47), as shown in Figure 5. After transforming Cognitive MSRF, the main effect of Phase was no longer significant (*F* (1.39) = 2.97, *p* = 0.09, $\eta_{\text{p}}^{2}$ = 0.07). The significant interaction between Knowledge manipulation X Phase reveals that participants in the Installation condition used significantly more cognitive MSRF when describing Exploitation phase compared to Context phase, whereas this difference was not observed in the Control condition.

**Figure 6:**
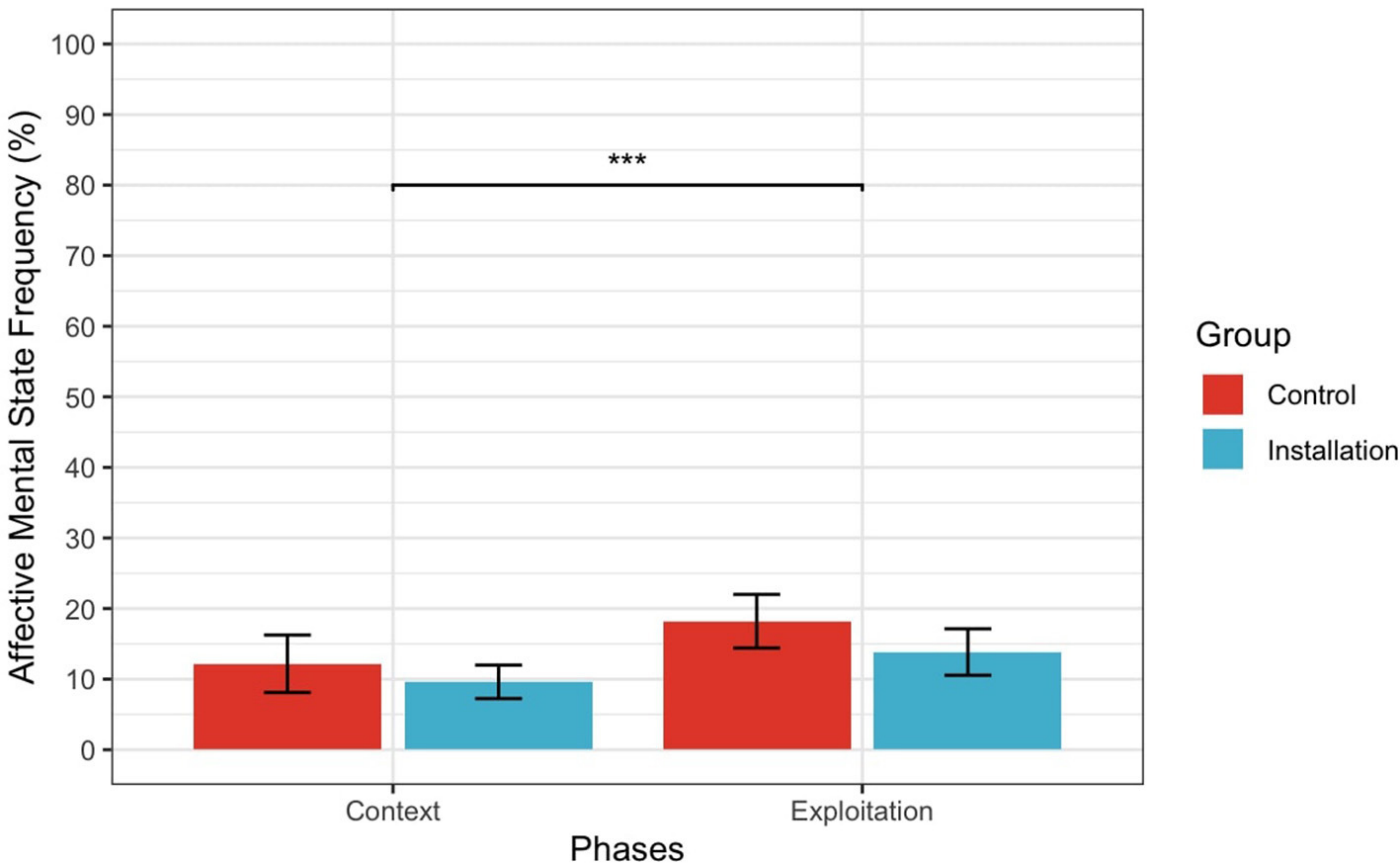
Interaction between Phase and Condition for Affective Mental State Frequency. Error bars represent 95% confidence intervals. ^*^Indicates a *p*-value less than 0.05, ^*^^*^Indicates a *p*-value less than 0.01, ^*^^*^^*^Indicates a *p*-value less than 0.001.

For H2.3 (Affective MSRF), there was a main effect of Phase (*F* (1.39) = 5.57, *p* < 0.05, $\eta_{\text{p}}^{2}$ = 0.30) but no significant main effect of Knowledge manipulation (*F* (1.39) = 3.14, *p* = 0.08, $\eta_{\text{p}}^{2}$ = 0.07) or interaction (*F* (1.39) = 0.53, *p* = 0.47, $\eta_{\text{p}}^{2}$ = 0.01). Importantly, these results, depicted in Figure 6, suggest that participants in the Installation condition did not use more Affective MSRF when describing Exploitation phase compared to Context phase, and neither did the Control participants.

### 3.4. Supplementary analysis examining relationship between DIcomp and MSRF

In the final step to understand the complementary relationship between the two dependent variables measured and their individual differences, we focused on the Installation group. We analyzed whether viewers who watched the installation scene and either partially or fully understood the dramatic irony conflict used higher Overall, Cognitive, and Affective MSRF in their descriptions across clips compared to those who failed to understand it. We also examined whether this varied depending on the Context or Exploitation phase for each clip.

To investigate this, we classified participants from the Installation group based on their DIcomp levels and performed a series of linear mixed effects models using the lme4 package (Bates et al., 2009). The results of these LME models are shown in Table 3. We used a random intercept linear mixed effects model, nesting six measurement occasions (one per clip) of MSRF (Overall, Cognitive, and Affective) within each participant and including the DIcomp scores (failed, partial, or full understanding) as categorical predictors and their interaction. While we considered the possible total scores for dramatic irony conflict comprehension (ranging from 0 to 12) for the manipulation check, in the LME models, each clip was given a score of 0, 1, or 2 separately, allowing us to maintain the categorical nature of comprehension levels while still enabling us to analyze the relationship between DI Comprehension and MSRF in a more nuanced manner. We built all models step by step to examine the effect of including the different terms in explaining mental state frequencies.

**Table 3:** Linear mixed effects models of the effect of dramatic irony comprehension on mental state reference frequency for the Installation group.

	**Overall MS references**			**Cognitive MS references**			**Affective MS references**		
**Predictors**	**Estimates**	**CI**	***p***	**Estimates**	**CI**	***p***	**Estimates**	**CI**	***p***
(Intercept)	24.07	11.39 – 36.76	**< 0.001**	18.99	5.64 – 32.34	**0.005**	7.31	−1.70 – 16.31	0.111
Partial DI comp	2.92	−15.26 – 21.10	0.752	3.66	−12.96 – 20.27	0.665	−4.10	−14.46 – 6.25	0.436
Full DI comp	13.70	0.20 – 27.21	**0.047**	7.58	−5.22 – 20.39	0.244	3.67	−4.29 – 11.62	0.365
Phase [Explo]	5.59	−11.23 – 22.41	0.514	1.53	−13.26 – 16.32	0.839	4.06	−5.44 – 13.56	0.401
Partial DI comp × Phase [Explo]	29.91	4.89 – 54.94	**0.019**	32.78	10.77 – 54.79	**0.004**	−2.87	−17.00 – 11.27	0.690
Full DI comp × Phase [Explo]	15.96	−2.30 – 34.23	0.086	15.32	−0.74 – 31.38	0.061	0.64	−9.67 – 10.96	0.902
**Random effects**									
σ^2^	619.72			479.19			197.64		
τ_00_	17.01 _Participant_num_			41.47 _Participant_num_			0.92 _Participant_num_		
	8.43 _Clip_			57.05 _Clip_			44.39 _Clip_		
N	21 _Participant_num_			21 _Participant_num_			21 _Participant_num_		
	6 _Clip_			6 _Clip_			6 _Clip_		
Observations	252			252			252		
Marginal *R*^2^/Conditional *R*^2^	0.216/0.247			0.170/0.312			0.056/0.232		

Bold values denote significance at the level *p* ≤ 0.05.

We included the variable “Clip” to check whether it was necessary to control for clip-level variables. The analysis for all DVs confirmed that adding “Clip” as a random intercept did not explain more variance and the fit of the model was worse for Overall MSRF (BIC: 2416.8 vs. 2411.3, *p* = 1); however, it was significant for Cognitive MSRF (BIC: 2364.8 vs. 2369.1, *p* = 0.002) and Affective MSRF (BIC: 2095.6 vs. 2118.4, *p* < 0.001). These results suggest that the measured DVs do vary across clips, indicating that some participants and clips tended to produce more MS references than others when considering the types of mental states independently, but this variation is balanced out when considering Overall MSFR.

**Figure 7:**
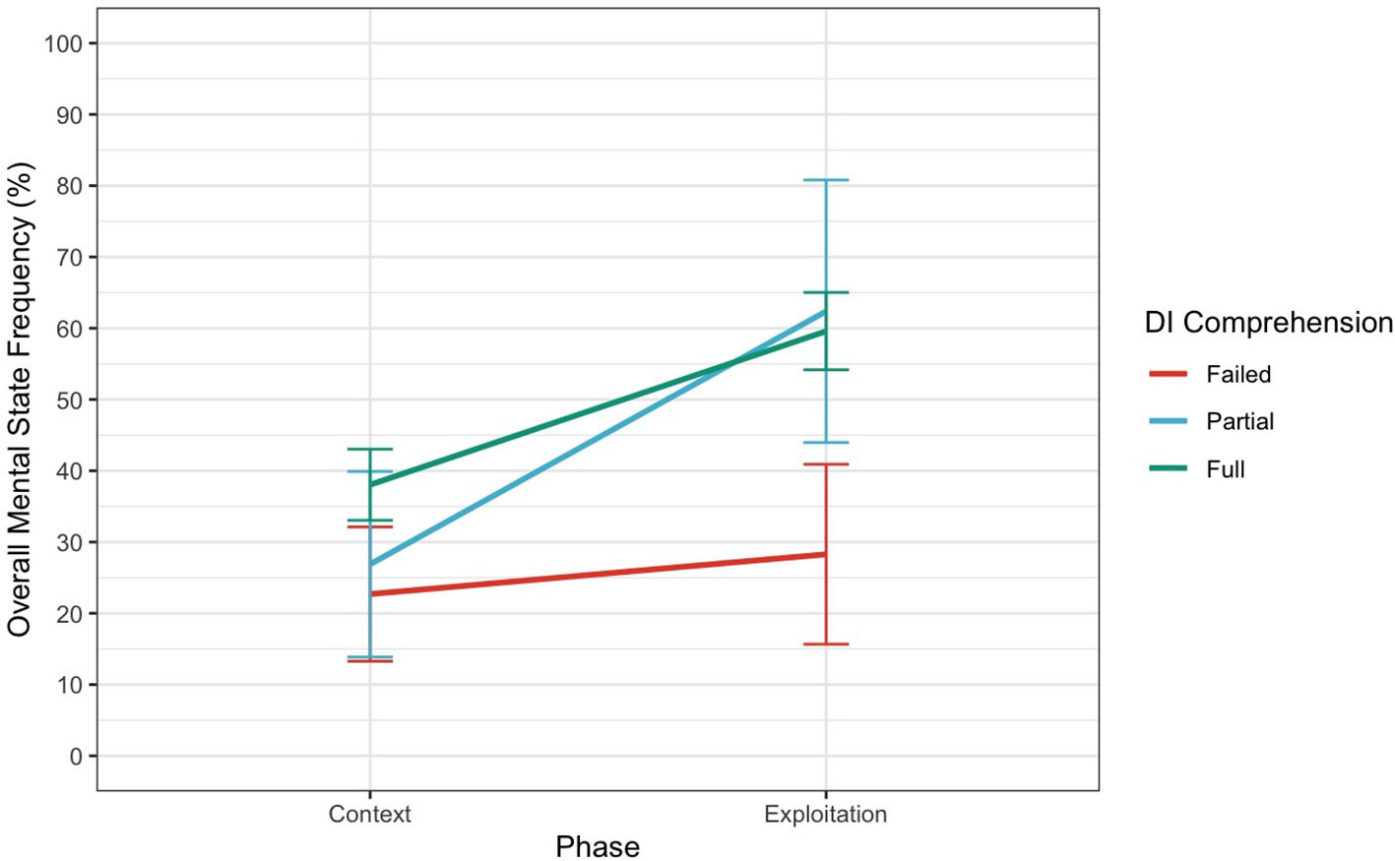
Interaction between Phase and DIcomp for Overall Mental State Frequency. Error bars represent 95% confidence intervals.

The results of the LME for Overall MSRF revealed a significant intercept (*p* < 0.001), indicating that participants used mental state references to describe the clips, even when they did not understand the DI conflict. Critically, the effect of DIcomp on Overall MSRF was not significant for partial DI comprehension (*p* = 0.752), but it was for full DI comprehension (*p* = 0.047), suggesting that fully understanding DI conflict can predict a higher Overall MSRF across the whole description. The main effect of Phase on Overall MSRF was not significant (*p* = 0.514). However, as depicted Figure 7, the interaction between DIcomp and Phase [Exploitation] indicated a significant increase in the use of Overall MSRF during the Exploitation phase for participants with partial DI comprehension (*p* = 0.019) but not for full DI comprehension (*p* = 0.086). This suggests that partially understanding the conflict can predict a higher use of Overall MSRF when focusing only on the Exploitation phase.

**Figure 8:**
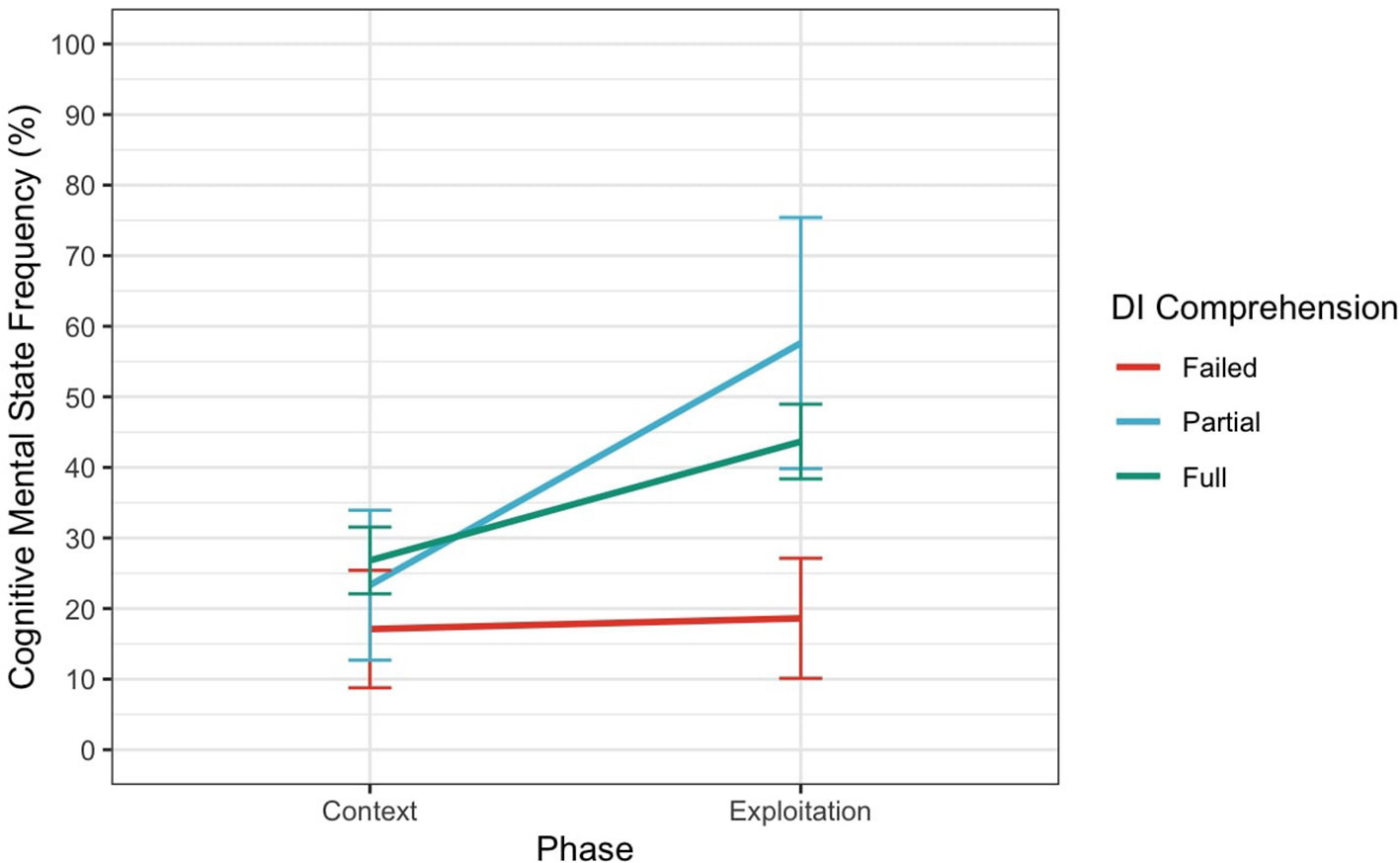
Interaction between Phase and DIcomp for Cognitive Mental State Frequency. Error bars represent 95% confidence intervals.

Similarly, the LME for Cognitive MSRF found a significant intercept (*p* = 0.005), indicating that participants in the Installation group used more Cognitive MSRF, on average, when describing the clips even when they showed failed understanding (see Figure 8). However, there was no significant effect of DIcomp on Cognitive MSRF. The main effect of Phase was not significant for Cognitive MSRF, but the interaction between DIcomp and Phase [Exploitation] was significant for Partial DIcomp (*p* = 0.004), suggesting that the use of mental state references was more frequent when participants were describing the Exploitation compared to the Context of the clips for those with partial DI comprehension but not with full DI comprehension (*p* = 0.061).

**Figure 9:**
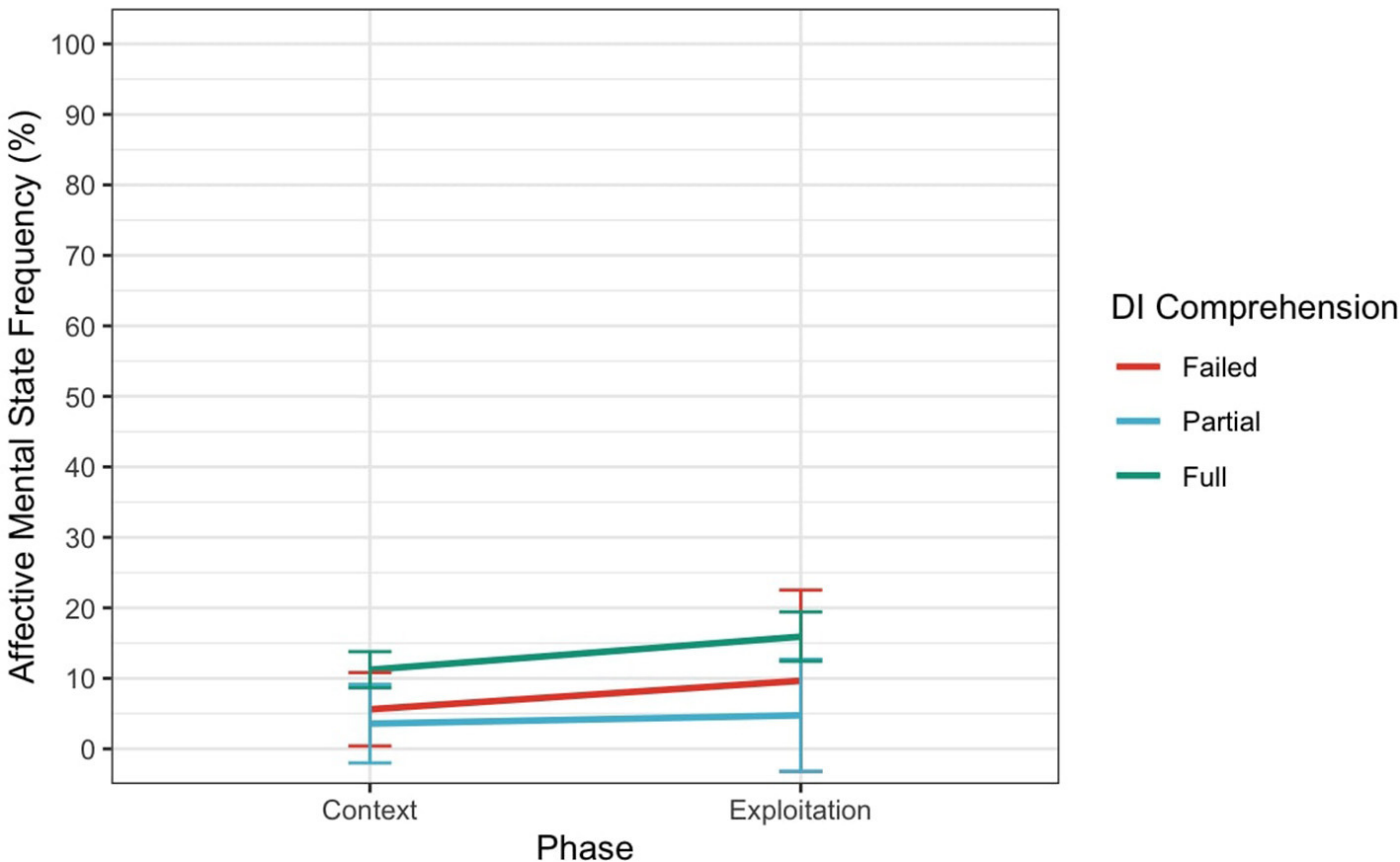
Interaction between Phase and DIcomp for Affective Mental State Frequency. Error bars represent 95% confidence intervals.

Lastly, the LME for Affective MSRF did not find a significant intercept (*p* = 0.111), indicating that participants exposed to the installation scenes did not use significant Affective MSRF, on average, in describing the clips. As depicted in Figure 9, the effect of DIcomp on Affective MSRF was not significant, and the main effect of Phase was also not significant for Affective MSRF. The non-significant interaction term suggests that the effect of the Exploitation phase on Affective MSRF is not significantly different for participants with failed comprehension scores from those with partial (*p* = 0.690) or full DI comprehension scores (*p* = 0.902).

## 4. Discussion

In this proof-of-concept study, we presented a novel approach to explore spontaneous theory of mind (SToM) using film structures. In particular, we used dramatic irony structures that prompt viewers to attribute ignorance and false beliefs to film characters. Our design allowed us to compare true- vs. false-belief spontaneous attribution scenarios in naturalistic settings. Our findings show that when participants watched the full dramatic irony clip with the *installation* scene, they understand the dramatic irony conflict more often than when they did not watch the scene, demonstrating that the exposure to this particular scene was required to understand dramatic irony.

The results showed that overall, participants in the Installation group used a higher Cognitive MSRF than the Control group but not a higher Affective MSRF, which remained similar across conditions. These results imply that salient divergence of knowledge in dramatic irony prompted participants to spontaneously refer to characters' epistemic mental states such as belief and knowledge, rather than affective mental states, in their character models.

Moreover, when controlling for content delivered and focusing on the scenes that both groups had seen, the Installation group used a higher Overall and Cognitive MSRF in their descriptions of the Exploitation phase than the Control group but showed no difference in the Context phase. While it was reasonable to expect participants in the Installation condition to use a high frequency of mental states to describe the Exploitation phase (given that this is where the dramatic irony conflict happens), it was also possible that these participants' event representation and retrospective recollection of the *establisher* scenes would be affected by the *installation* scene and dramatic irony conflict, prompting them to use a high frequency of mental states in the description of the Context phase. This was not the case. The present findings suggest that the salient disparity of knowledge in dramatic irony scenes prompts viewers to infer the cognitive mental states of characters and specifically when they access and retrieve event models of the *exploitation* scenes. In this regard, we also recognize that participants in the Installation condition had more events and characters to describe, which may have led to fatigue. However, if fatigue was to play a significant role, we would expect the Installation group to engage less with the mental states of characters, as the literature suggests that fatigue can negatively impact social problem-solving and empathic responding (Nelson et al., 2003; Nelson, 2018). This hypothesis would lead us to anticipate the opposite pattern of results to what we have found in our study. Moreover, given this fatigue bias, we might expect the Control group, who had less content to describe in the context scenes compared to the Installation group, to engage more with the mental states of characters due to the lack of fatigue. Yet, our findings do not support this prediction.

Finally, we investigated individual differences in DI comprehension in those participants that had watched the complete dramatic irony version and how this comprehension relates to mental state reference frequency. Our findings suggest that by default, participants used Overall and Cognitive but not Affective MSRF to describe the clips, even when they failed to understand the DI conflict. Moreover, we found that Overall MSRF increased with full understanding of DI conflict. Interestingly, partial understanding of DI conflict also predicted higher Overall and Cognitive MSRF (but not Affective MSFR) when participants described the Exploitation phase vs.s when they described the Context phase. Our findings suggest that participants tended to use a similar amount of cognitive and affective mental state terms when they watched scenes where they knew the same amount of information as the characters (as indicated by the significant intercepts in a second set of LMEs carried out for the Control group; see Supplementary material for this table). However, when participants were exposed to critical knowledge that a character did not possess, they describe the event in terms of their cognitive mental states, but they do not significantly refer to their affective mental states. Interestingly, this was the case even when they do not show understanding of dramatic irony. This suggests that the extent to which we focus on affective vs. cognitive mental states in describing a scene depends on our level of knowledge relative to the characters in the scene.

Taken together, these findings highlight the importance of differentiating between cognitive and affective mental states when measuring SToM through mental state reference frequencies. Participants use different frequencies of these two types of mental states depending on their comprehension, i.e., on the event model they built of the situation, thus implying that their differentiation is key for our understanding of the nature of SToM responses in dramatic irony scenes. This perspective aligns with the work of authors such as Shamay-Tsoory and Aharon-Peretz (2007), who found evidence for an anatomical distinction between affective and cognitive ToM processing, suggesting that they rely on partially separate anatomical substrates.

Similar to how verbal irony paradigms have been extensively used to probe the relationship between language processes and theory of mind and their individual differences (e.g., Hancock et al., 2000; Filippova and Astington, 2008; Nilsen et al., 2011; Pexman et al., 2011), dramatic irony in film can constitute a powerful tool for investigating social cognition processes in the domain of visual event perception and comprehension. Moreover, the present dramatic irony film corpus offers a unique opportunity to examine the underlying sociocognitive processes involved in event perception and comprehension in several ways.

First, it can allow us to examine whether and how individuals attribute knowledge or epistemic states in the *installation* scenes of dramatic irony. In particular, when labeling characters as ignorant, we attribute them a lack of knowledge due to either lack of perceptual access or due to other contextual factors such as repression, denial, or mental illnesses (Lavandier, 2005). Traditional ToM stimuli such as the Sally–Anne task and dramatic irony in film have in common that they both clearly cue lack of perceptual access; however, they achieve this in different ways. The Sally–Anne task manifestly shows a character leaving a room to signpost their lack of presence in the scene, while dramatic irony, which could be considered a cinematic version of the mentioned task, implies who has access to information through subtler means, embedded in a continuous, intricate narrative The complexity of ToM reasoning in these clips, as seen in the descriptions contained in Table 1, is much greater compared to the relatively straightforward Sally–Anne task. To understand who is ignorant in these scenes, viewers are inadvertently motivated to track knowledge as part of the broader event sequence. In silent films like the Harold Lloyd films chosen for our film corpus, long or medium-long shots were often used due to the composition trends of the time, where people walked in and out of the scenes similar to theater performances. In modern films, knowledge is indicated through cinematic techniques which are familiar to the viewer, such as flashbacks scenes, camera cutaways to different locations, point-of-view shots, or carefully choreographed staging in which character knowledge of foreground events may differ to background events. Other cinematic techniques include editing, which can shape the audience's experience of the narrative, and lighting, focusing, or camera movements, which can be used to direct the audience's attention to specific details within a shot.

Despite the absence of color and sound, as well as the presence of intertitles in these clips, the visual storytelling in these silent films relies heavily on facial expressions, body language, and context, which are essential components of real-life social interactions. These elements allow viewers to make inferences about characters' mental states, intentions, and emotions, thus providing a valuable stimulus for studying ToM processing. Additionally, while film editing techniques may not be naturalistic representations of reality, they do serve to guide and shape viewers' cognitive processes, allowing for the examination of how these cinematic choices influence ToM processing (Cabañas et al., 2022; Grall and Finn, 2022). In fact, the stylized nature of the stimulus may help to focus participants' attention on specific aspects of the narrative and ToM processing, allowing us to isolate these processes to study them with a degree of ecological validity.

It is essential to consider potential limitations in applying these findings to real-life social interactions due to unique cinematic elements and differing participant identification or empathy with characters. Nevertheless, film viewing offers a valuable opportunity to study cognitive processes, as it allows for spontaneous processing in a controlled environment that is challenging to achieve in real-world scenarios or virtual reality, where participants have a unique experience as they choose what is within their field of view at any moment. This controlled setting allows for a more uniform investigation of mental processes among participants. Nonetheless, future research should examine the relevance of our findings to real-world contexts and the potential limitations of generalizing results from film viewing to real-life social interactions.

Second, the knowledge manipulation in these clips seeks to address scenarios that induce either false beliefs (in the Installation condition) or true beliefs (in the Control condition), thereby creating differences in the divergence in the interpretations of the shared content (the Exploitation phase) based on prior knowledge, allowing us to compare how viewers process the same events depending on their false- or true-belief representations. In addition, this comparison speaks to the debate of automaticity and modularity in belief attribution [e.g., (Fodor, 1992; Leslie and Thaiss, 1992; Back and Apperly, 2010)]. Critically, the current findings suggest that false-belief inducing scenarios prompt a richer cognitive mental state representation than true-belief inducing scenarios accessed by participants in their free recall answers. In line with these results which suggest increased complexity of false-belief representations, there is evidence that adults are slower making judgements when a character had a false belief rather than a true belief (German and Hehman, 2006). Moreover, Phillips et al. (2011) found that while elderly adults perform similar to young adults on true-belief tasks, they perform worse in false-belief tasks. However, the mentioned studies often use explicit and repetitive ToM paradigms, which, as suggested previously, may not capture the full picture of everyday ToM processing. Employing measures that address cognitive effort to compare the sociocognitive processing of both dramatic irony clip versions could allow us to understand better whether and how false beliefs are more cognitively effortful than true beliefs in spontaneous mentalizing in a more ecologically valid context.

Note that the current results showed room for refinement for our research paradigm, in particular the choice of film stimuli. Notably, there was variation in the DI conflict comprehension scores, specifically for Clip 1, which resulted in no significant differences in DI comprehension between the groups, and Clip 5, which was similarly not well-understood in both groups. In Clip 1, formal cinematic cues may have helped participants understand dramatic irony even without the installation scene, while the absence of such cues in Clip 5 may have hindered its comprehension. Additionally, our coding scheme required participants to identify both ignorance and consequences for the victims of dramatic irony. However, we observed a “protagonist effect,” where descriptions focused on the protagonist even when they were not the victim of dramatic irony, leading to insufficient criteria for DI comprehension. This is similar to Hutson et al. (2017), who found the “agent effect,” where eye movements appear to be influenced by whether viewers perceive a character in the narrative as an agent or just a character who appears in the background without playing a significant role. Addressing these issues is crucial for appropriate comparison between Installation and Control conditions.

A potential limitation to address is that, precisely due to the expected intrinsic power of dramatic irony to motivate participants to attribute mental states to characters, we expected a certain overlap between comprehension of DI conflict and mental state scores. While both measures involve language-based descriptions of mental states, they are distinct constructs that do not necessarily depend on each other. Moreover, note that the coding scheme, based on Barnes and Baron-Cohen (2012) and the definition of dramatic irony conflict by professional scriptwriter theorist Lavandier (2005), did not require participants to use mental states to be scored as partial and full understanding. It is possible for participants to have a good understanding of the DI conflict without necessarily mentioning the victim's mental states in their descriptions. An example of a description scoring partial understanding without using mental state words is “the publisher exchanges the rejection letter for a check at the last moment.” A description demonstrating full without mental state words might be “*Harold rips up the envelope containing the cheque instead of a rejection letter*.” Conversely, a participant might use numerous mental state references without necessarily having a good understanding of the dramatic irony conflict. For instance, answers such as “*Harold was very disappointed at himself, he felt a failure as a writer*” would not be awarded any points. Moreover, in the present study, we found that participants produced higher Overall and Cognitive MSRF when they partially understood the conflict than when they fully understood it, remarking that DIcomp and MSRF do not co-vary linearly, speaking to the fact that these measures address different aspects of dramatic irony processing.

In short, while there may be certain overlap between the comprehension measures and mental state references frequency, the results of the present study suggest that these are distinct constructs that do not necessarily depend on each other. The DIcomp measure provides valuable insights into individual differences in the ability to comprehend scenes and can be particularly useful in investigating the cognitive and affective processes involved in this type of scene comprehension. On the contrary, the frequency of mental state references demonstrates the extent to which individuals integrate mentalistic attributions into their character models. Although this often results in inferences about the consequences of false beliefs, it is not a strict requirement for comprehending the DI conflict. By examining both the understanding of the DI conflict and the frequency of mental state references, we obtain a more nuanced multi-dimensional of how SToM is involved in the comprehension of dramatic irony.

We recognize the potential influence of individual differences, especially in verbal abilities, on mental state descriptions. Although we did not explicitly control for verbal abilities, all participants possessed university-level English proficiency, establishing a baseline for language skills and minimizing the impact of individual differences. To further account for individual differences in verbal production, we adjusted for the length of participants' descriptions by dividing the number of mental state references by the number of coding units. Notably, differences in mental state references were observed within subjects when comparing Context and Exploitation phases, which mitigates the potential influence of individual differences in verbal abilities on our results. In future research, we plan to address individual differences more specifically, such as atypical theory of mind skills as it is common in ASC, and verbal abilities that may impact mental state descriptions, to better understand their role in our findings and enhance the generalizability of our results.

In future studies, the present dramatic irony film corpus and measures could be used in conjunction with other measures of SToM, such as eye tracking, psychophysiological monitoring of affective states and arousal, or functional neuroimaging during the processing of dramatic irony. This would enable researchers to identify individual differences in mentalizing processes, affective states, and help isolate neural structures responsible in the moment-to-moment false- vs. true-belief spontaneous attribution, such as the much-debated involvement of the medial prefrontal cortex (mPFC) in SToM (Bardi et al., 2016; Moessnang et al., 2017; Boccadoro et al., 2019). As already mentioned, these narrative devices are pervasive in cinema; therefore, researchers can identify these structures in open datasets to take advantage of free-viewing SToM paradigms (Eickhoff et al., 2020).

In concurrent work with this film corpus, we are exploring the role of supporting cognitive processes of ToM, such as working memory and attention, in the comprehension of dramatic irony. By examining the interplay between SToM and these cognitive processes, we aim to provide a more comprehensive understanding of the factors contributing to the successful processing and appreciation of dramatic irony in narrative contexts. By examining changes in eye movements and neural activity during the processing of the present dramatic irony film corpus, researchers could gain a better understanding of how SToM operates in real time during social interactions. On this note, we believe it is important to highlight that eye tracking, physiological, and functional neuroimaging techniques proposed should be used in conjunction with comprehension measures as the ones presented. As stated in Cabañas et al. (2022), simply investigating eye movements or brain activity without using additional comprehension measures to correlate with the observed brain response should not be viewed as conclusive evidence of viewers' cognitive representations, thereby constituting a type of reverse inference (Poldrack, 2006).

Moreover, our dramatic irony film corpus could be employed in research investigating developmental differences in SToM processing, by comparing how children, adolescents, and adults process and understand dramatic irony. This line of research would provide valuable insights into the development of mentalizing skills and social understanding across different age groups. The stimuli, procedures, and coding handbook used in our study are available upon request, making it accessible for researchers interested in further exploring this topic.

Lastly, our film corpus and measures could be adapted to examine the efficacy of interventions aimed at improving atypical ToM processing, such as in individuals with ASC. By using the film corpus as a tool to measure the effectiveness of these interventions, researchers can assess the real-world applicability of the developed strategies and their potential for improving social understanding in everyday life.

## 5. Conclusion

The implications of the present proof-of-concept study are three-fold. First, it demonstrates that the degree to which individuals emphasize affective vs. cognitive mental states is influenced by their level of knowledge relative to that of the characters in the scene. Second, our study highlights the utility of a novel corpus of dramatic irony film scenes as a means of investigating social cognition in ecologically valid contexts, enabling us to address knowledge, true- and false-belief attributions. Third, we provide measures of comprehension and mental state attribution, which address complementary aspects of social processing in scene perception and event comprehension, essential to allow the exploration of links between different levels of cognitive processing and eye tracking or neural dynamics. The integration of these measures with other techniques could have important implications for our understanding of moment-to-moment SToM and the neural underpinnings of social processing.

## Data availability statement

The raw data supporting the conclusions of this article will be made available by the authors, without undue reservation.

## Ethics statement

The studies involving human participants were reviewed and approved by Birkbeck, University of London Ethics Board (181949). The patients/participants provided their written informed consent to participate in this study.

## Author contributions

CC, AS, and TS contributed to conception and design of the study. CC collected the data, performed the statistical analysis, and wrote the first draft of the manuscript. All authors contributed to manuscript revision and read and approved the submitted version.
