# Supplementary material for: The audience who knew too much: investigating the role of spontaneous theory of mind on the processing of dramatic irony scenes in film
Source: Front Psychol. 2023 Jul 4;14:1183660. doi: 10.3389/fpsyg.2023.1183660 (PMC10353302; doi:10.3389/fpsyg.2023.1183660)
Supplement: Supplementary file 1 [file Data_Sheet_1.docx]

Supplementary Material

The Audience Who Knew Too Much: Investigating the Role of Spontaneous Theory of Mind on the Processing of Dramatic Irony Scenes in Film

Cynthia Cabañas^1*^, Atsushi Senju^1,2^, Tim J Smith^1^

*** Correspondence:** Cynthia Cabañas: ccaban01@mail.bbk.ac.uk

Supplementary Data

## Description of Clips

**Clip 1 - Never Weaken Part 1:**

*Establisher*: Harold wants to help his friend or love interest, who will get fired from the osteopathic clinic where she works if they do not get more clients.

*Installation*: Harold runs into an acrobat and they come up a plan together.

*Exploitation*: The acrobat pretends to fall and get injured in front of a crowd of potential clients and Harold pretends to be an osteopath who can help him. When the acrobat seems to heal quickly, the crowd gets excited and asks for business cards for the osteopathic clinic.

*Victim of dramatic irony*: the people in the crowd whose goal is to get a treatment for their illnesses or disabilities.

**Clip 2 - The Freshman:**

*Establisher*: Harold out for the team to be a football player

*Installation*: After tryouts, Harold is unaware that he did not make the team. The coach and teammate trick him into thinking he made the team but assign him the role of water boy.

*Exploitation*: Harold excitedly tells a girl that he made the team and eagerly goes to the field to play because he thinks he is on the team, unaware that he is really just the water boy.

*Victim of dramatic irony*: Harold whose goal is to be a football player.

**Clip 3 - Never Weaken Part 2:**

*Establisher*: Harold proposes to a girl, and she accepts.

*Installation*: In another scene, the girl is talking to a man who is actually her brother, and he's an ordained minister.

*Exploitation*: Harold overhears the man offering to marry the girl, not realizing that he's actually her brother. Harold thinks the man wants to marry her, not to officiate the wedding, so he's visibly upset and walks away.

*Victim of dramatic irony*: Harold whose goal is to marry the girl.

**Clip 4 - Girl Shy:**

*Establisher*: Harold visits a publishing house to inquire about the possibility of publishing his book. However, the publisher finds his book to be extremely comical, so they reject it and inform Harold that he will receive a rejection letter in the mail.

*Installation*: When Harold leaves, a senior employee convinces the editor to reconsider and publish the manuscript as a comedy. He then instructs the employee to send a check to Harold instead of the rejection letter.

*Exploitation*: Harold, downhearted and unaware of the content of the letter, tears it apart without opening it.

*Victim of dramatic irony*: Harold whose goal is to publish his book and make money with it.

**Clip 5 - For Heaven’s Sake:**

*Establisher*: A missionary and his daughter need to raise money for a homeless mission and decide to write a letter to Harold, who is portrayed as a wealthy man.

*Installation*: Harold accidentally burns down a cart that belongs to the missionary.

*Exploitation*: Harold wants to pay for the burnt cart, so he writes a check for the missionary, who doesn't know that Harold caused the accident. The missionary and his daughter are grateful and think that Harold made the donation on purpose to help the mission.

*Victim of dramatic irony*: the missionary and his daughter, whose goal is to raise money for the mission.

**Clip 6 - The Kid Brother:**

*Establisher*: Two men running a traveling show are seeking a permit from the sheriff.

*Installation*: Harold feels inferior to his brothers and is not allowed to go to town with them. He stays at home and dresses up as his father, who is the sheriff.

*Exploitation* The two men see Harold dressed as sheriff and convince him to sign the permit.

*Victim of dramatic irony*: The two men, whose goal is to get the permit for their show.

# Supplementary Figures and Tables

For more information on Supplementary Material and for details on the different file types accepted, please see [here](https://www.frontiersin.org/guidelines/author-guidelines#supplementary-material).

|  | **Total MS References** | | | **Cognitive MS References** | | | **Affective MS References** | | |
| --- | --- | --- | --- | --- | --- | --- | --- | --- | --- |
| *Predictors* | *Estimates* | *CI* | *p* | *Estimates* | *CI* | *p* | *Estimates* | *CI* | *p* |
| (Intercept) | 34.07 | 25.50 – 42.64 | **<0.001** | 22.93 | 16.62 – 29.25 | **<0.001** | 10.79 | 2.97 – 18.60 | **0.007** |
| DIcomp [1] | 0.11 | -25.14 – 25.36 | 0.993 | -3.19 | -24.01 – 17.63 | 0.763 | 5.08 | -11.61 – 21.76 | 0.549 |
| DIcomp [2] | 19.36 | 3.72 – 35.00 | **0.015** | 11.89 | -0.92 – 24.69 | 0.069 | 9.67 | -0.90 – 20.24 | 0.073 |
| Phase [Exploitation] | -0.24 | -7.33 – 6.85 | 0.946 | -7.99 | -13.90 – -2.07 | **0.008** | 7.74 | 3.14 – 12.35 | **0.001** |
| DIcomp [1] × Phase [Exploitation] | 28.60 | -4.65 – 61.85 | 0.091 | 37.96 | 10.23 – 65.70 | **0.008** | -9.36 | -30.97 – 12.24 | 0.394 |
| DIcomp [2] × Phase [Exploitation] | -11.74 | -31.24 – 7.75 | 0.237 | -2.29 | -18.55 – 13.97 | 0.781 | -9.45 | -22.12 – 3.22 | 0.143 |
| **Random Effects** | | | | | | | | | |
| σ^2^ | 679.99 | | | 473.05 | | | 287.06 | | |
| τ_00_ | 135.10 _Participant_num_ | | | 49.27 _Participant_num_ | | | 23.42 _Participant_num_ | | |
|  | 33.83 _Clip_ | | | 19.23 _Clip_ | | | 69.89 _Clip_ | | |
| N | 21 _Participant_num_ | | | 21 _Participant_num_ | | | 21 _Participant_num_ | | |
|  | 6 _Clip_ | | | 6 _Clip_ | | | 6 _Clip_ | | |
| Observations | 252 | | | 252 | | | 252 | | |
| Marginal R^2^ / Conditional R^2^ | 0.044 / 0.234 | | | 0.079 / 0.195 | | | 0.039 / 0.275 | | |

**Supplementary Table 1**. Linear Mixed Effects models of the effect of dramatic irony comprehension on mental state reference frequency for the Control group.

Supplementary Figures
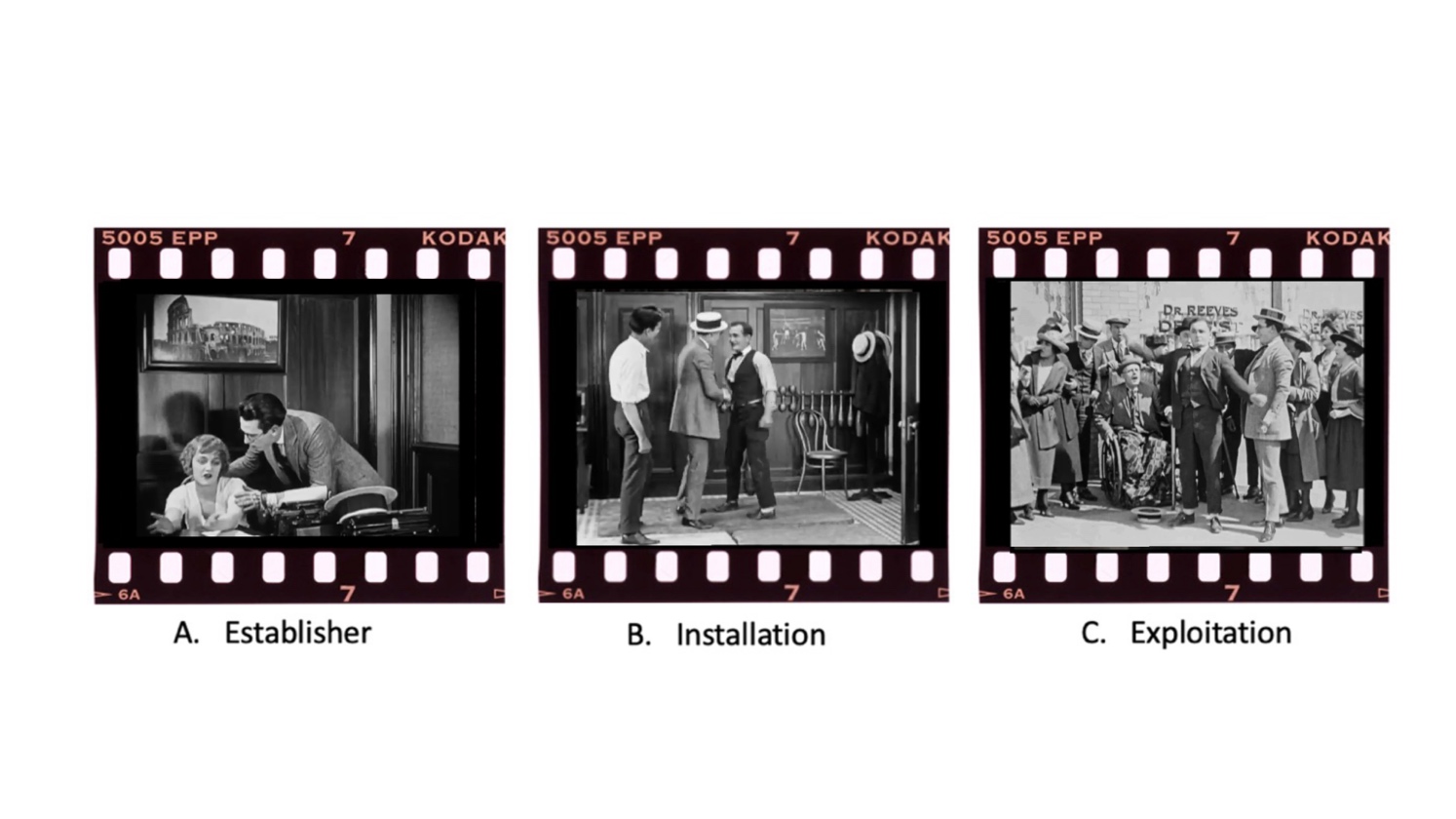
**Supplementary Figure 1.** Example stills from Clip 1. Stills taken with permission from Never Weaken (Copyright of the Harold Lloyd Trust, 1921). Reproduced with permission.

**
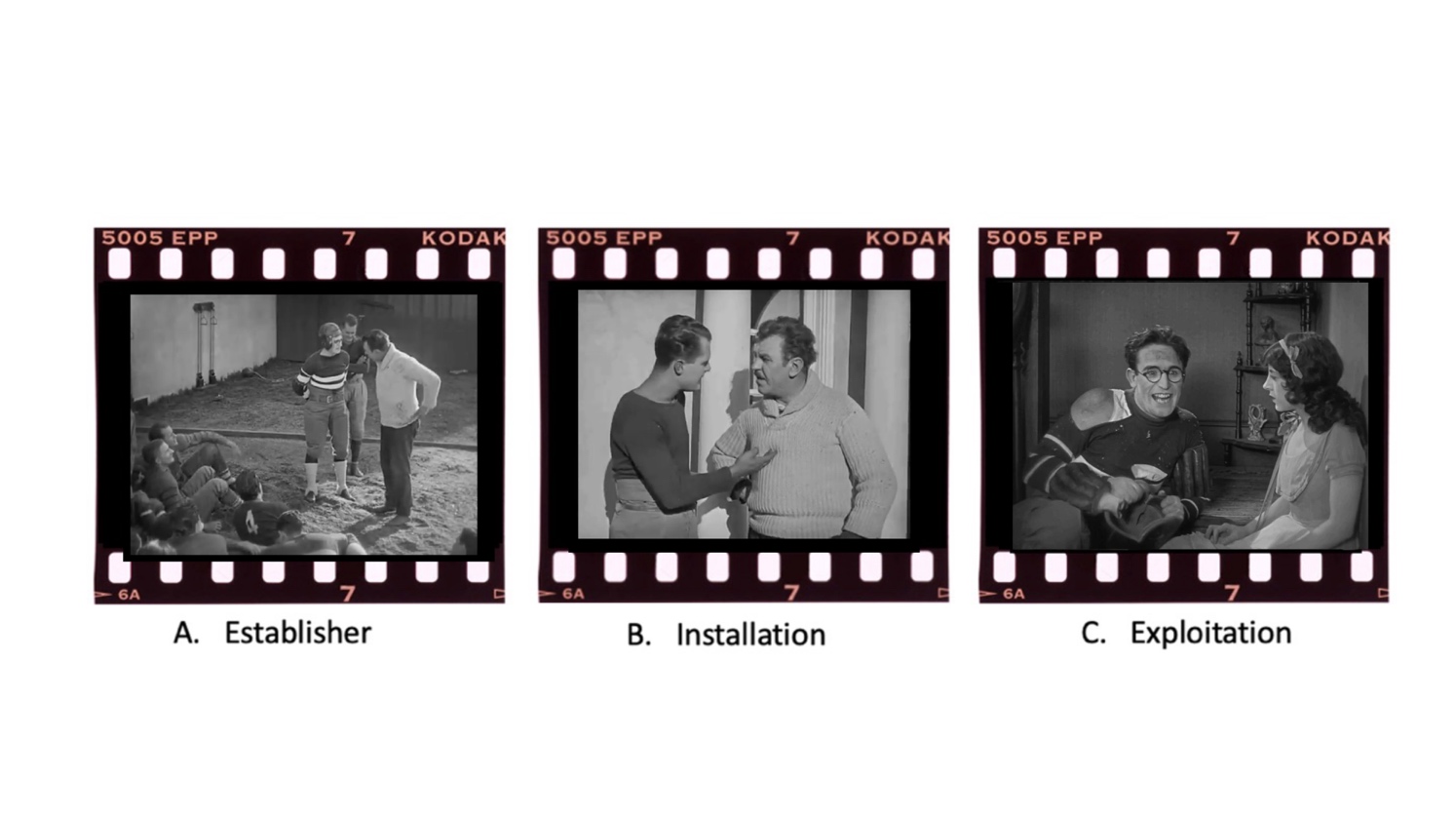
Supplementary Figure 2.** Example stills from Clip 2. Stills taken with permission from The Freshman (Copyright of the Harold Lloyd Trust, 1925). Reproduced with permission.

**
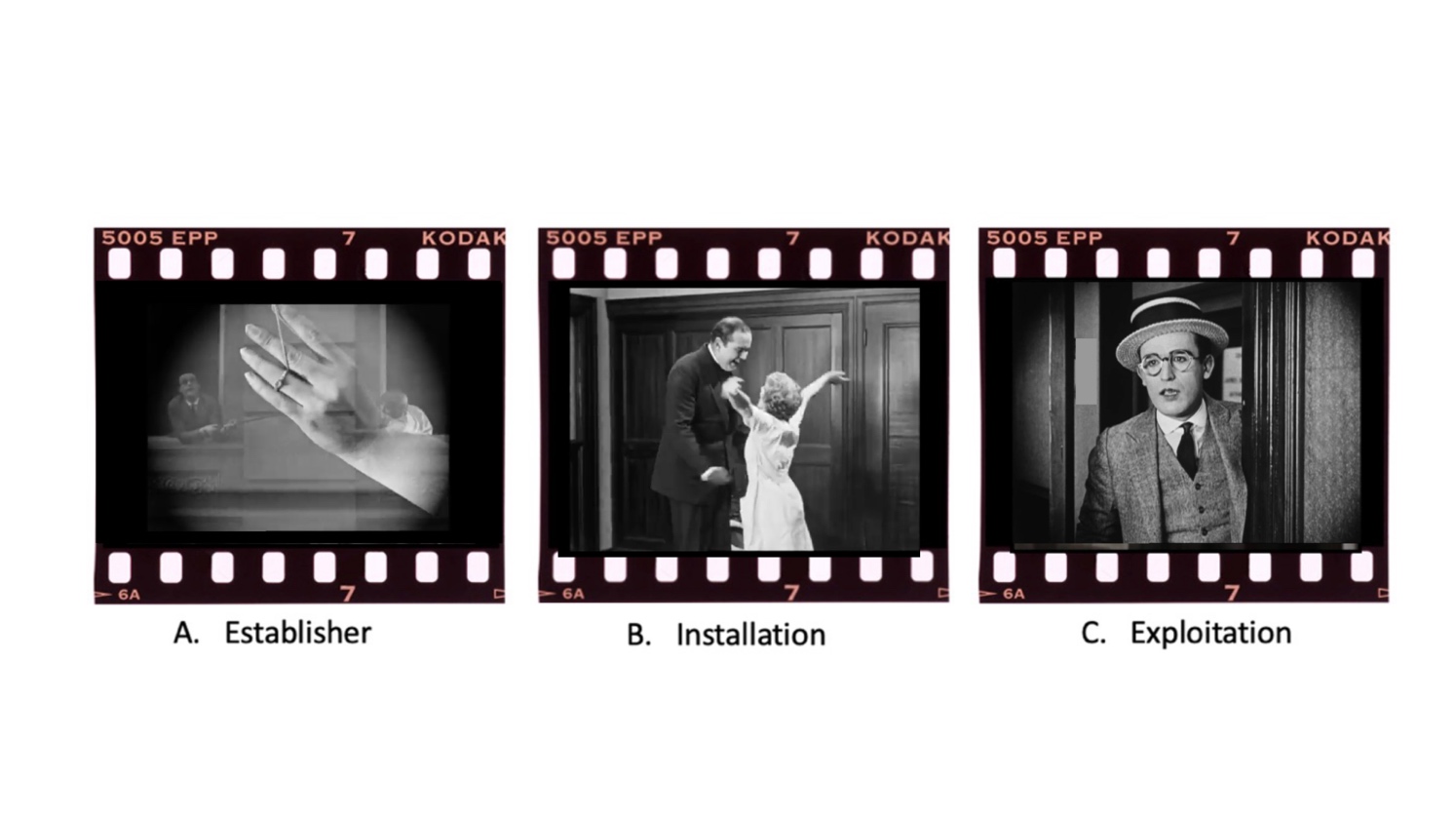
Supplementary Figure 3.** Example stills from Clip 3. Stills taken with permission from Never Weaken (Copyright of the Harold Lloyd Trust, 1921). Reproduced with permission.

**
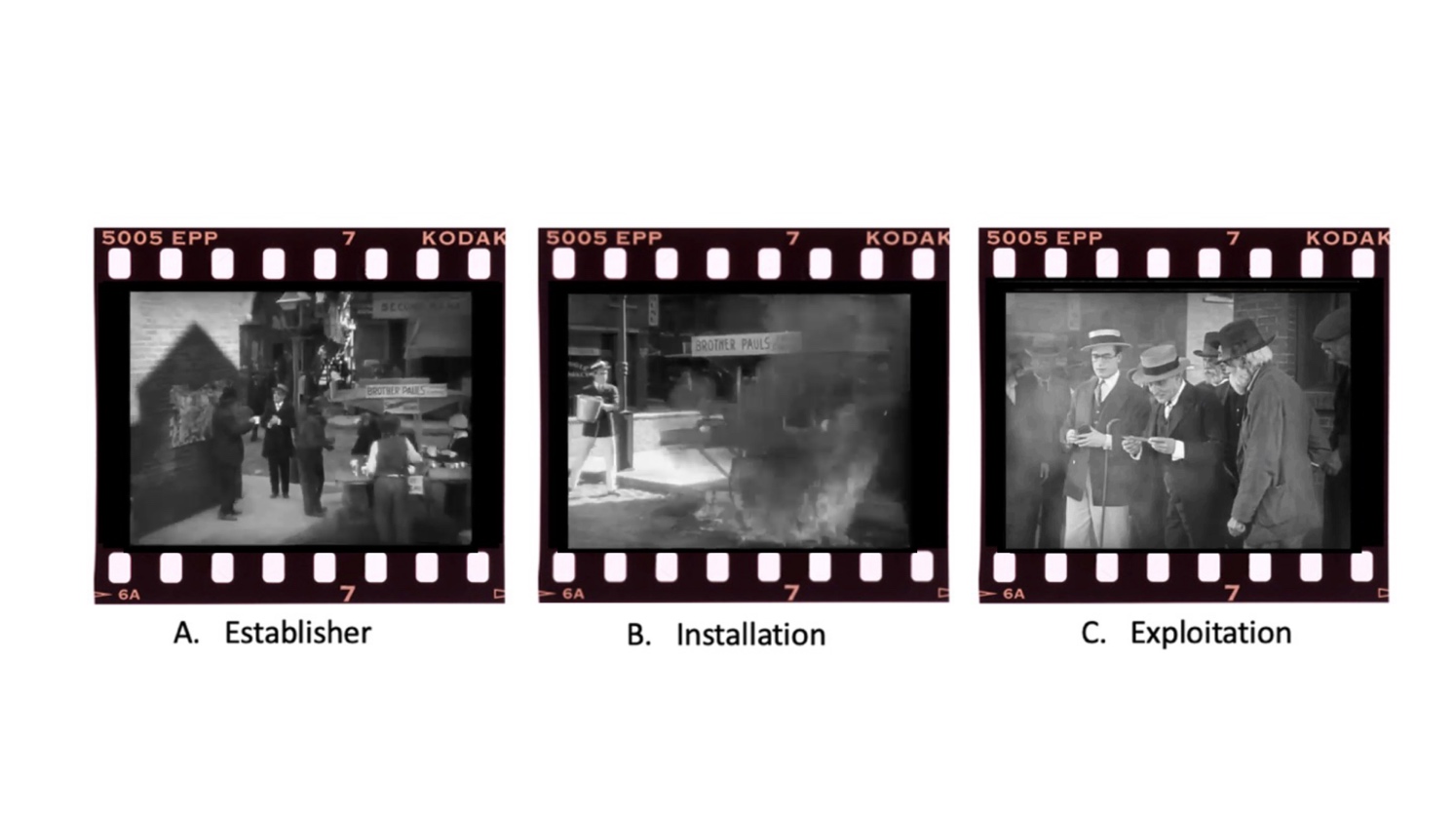
Supplementary Figure 5.** Example stills from Clip 5. Stills taken with permission from For Heaven’s Sake (Copyright of the Harold Lloyd Trust, 1926). Reproduced with permission.

**
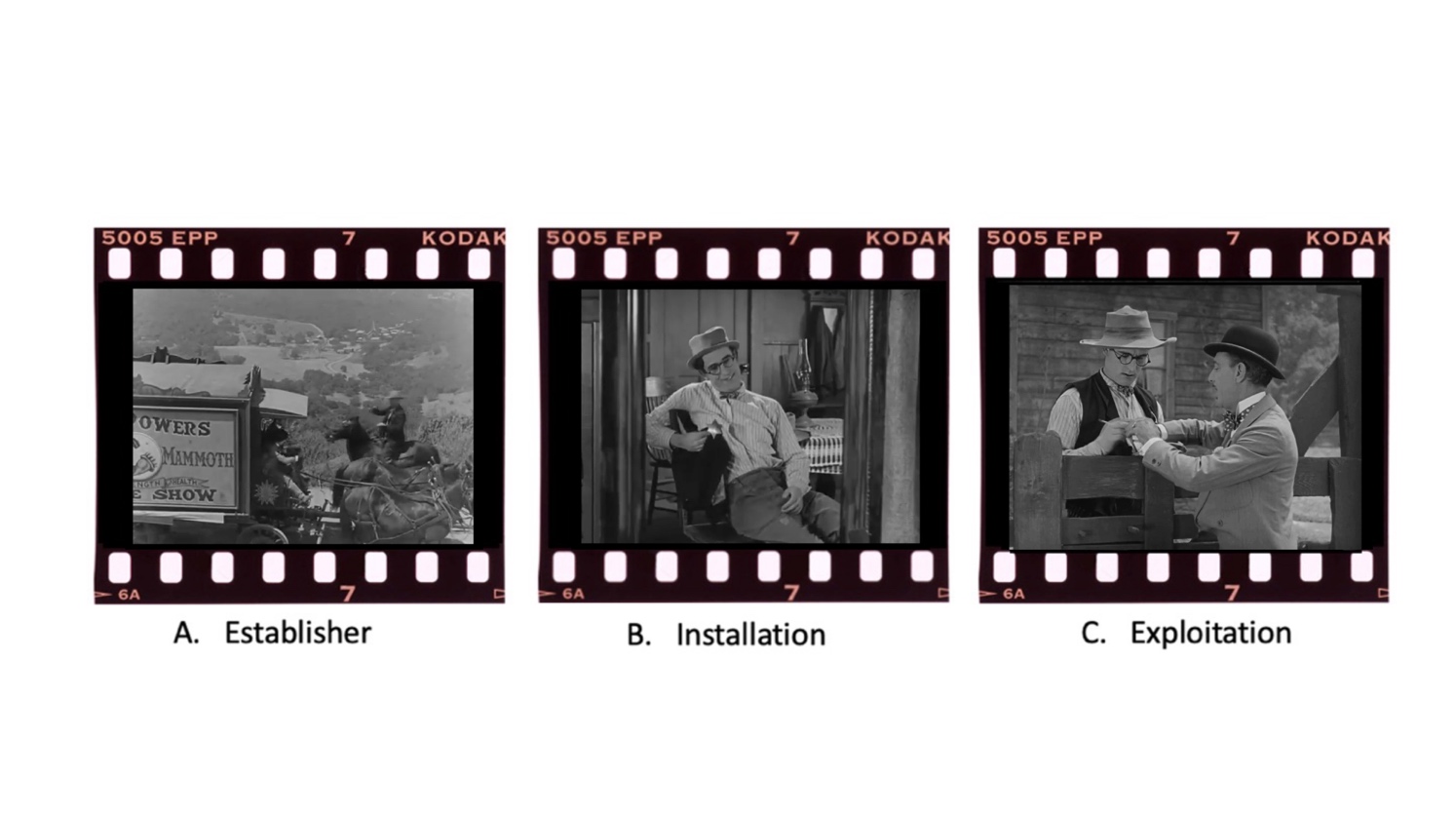
Supplementary Figure 6.** Example stills from Clip 6. Stills taken with permission from The Kid Brother (Copyright of the Harold Lloyd Trust, 1927). Reproduced with permission.


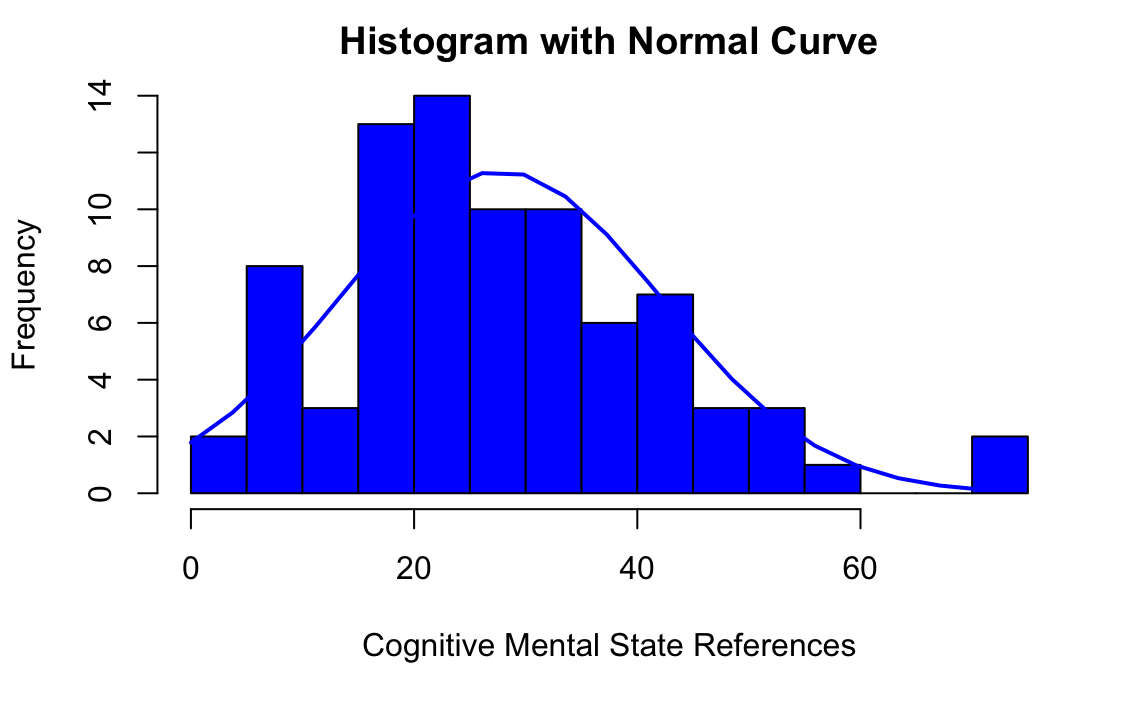


**Supplementary Figure 7.** Histogram for Cognitive Mental State Reference Frequency.
